# Functional characterization of a highly specific l-arabinose transporter from *Trichoderma reesei*

**DOI:** 10.1186/s12934-021-01666-4

**Published:** 2021-09-08

**Authors:** Sami Havukainen, Jonai Pujol-Giménez, Mari Valkonen, Matthias A. Hediger, Christopher P. Landowski

**Affiliations:** 1 VTT Technical Research Center of Finland Ltd, Tietotie 2, 02150 Espoo, Finland; 2 Membrane Transport Discovery Lab, Department of Nephrology and Hypertension, University of Bern, Freiburgstrasse 15, 3010 Bern, Switzerland; 3 Department of Biomedical Research, Inselspital, University of Bern, Freiburgstrasse 15, 3010 Bern, Switzerland

**Keywords:** *Trichoderma reesei*, Transmembrane transport, Arabinose transporter, Pentose fermentation

## Abstract

**Background:**

Lignocellulose biomass has been investigated as a feedstock for second generation biofuels and other value-added products. Some of the processes for biofuel production utilize cellulases and hemicellulases to convert the lignocellulosic biomass into a range of soluble sugars before fermentation with microorganisms such as yeast *Saccharomyces cerevisiae*. One of these sugars is l-arabinose, which cannot be utilized naturally by yeast. The first step in l-arabinose catabolism is its transport into the cells, and yeast lacks a specific transporter, which could perform this task.

**Results:**

We identified Trire2_104072 of *Trichoderma reesei* as a potential l-arabinose transporter based on its expression profile. This transporter was described already in 2007 as d-xylose transporter XLT1. Electrophysiology experiments with *Xenopus laevis* oocytes and heterologous expression in yeast revealed that Trire2_104072 is a high-affinity l-arabinose symporter with a *K*_m_ value in the range of $\sim$ 0.1–0.2 mM. It can also transport d-xylose but with low affinity (*K*_m_ $\sim$ 9 mM). In yeast, l-arabinose transport was inhibited slightly by d-xylose but not by d-glucose in an assay with fivefold excess of the inhibiting sugar. Comparison with known l-arabinose transporters revealed that the expression of Trire2_104072 enabled yeast to uptake l-arabinose at the highest rate in conditions with low extracellular l-arabinose concentration. Despite the high specificity of Trire2_104072 for l-arabinose, the growth of its *T. reesei* deletion mutant was only affected at low l-arabinose concentrations.

**Conclusions:**

Due to its high affinity for l-arabinose and low inhibition by d-glucose or d-xylose, Trire2_104072 could serve as a good candidate for improving the existing pentose-utilizing yeast strains. The discovery of a highly specific l-arabinose transporter also adds to our knowledge of the primary metabolism of *T. reesei*. The phenotype of the deletion strain suggests the involvement of other transporters in l-arabinose transport in this species.

**Supplementary Information:**

The online version contains supplementary material available at 10.1186/s12934-021-01666-4.

## Background

Lignocellulose biomass has gained considerable attention as a feedstock for bioprocesses. It is renewable and abundant all over the world, and its utilization as a feedstock does not interfere with food production, as is the case for the starch containing feedstocks used to create 1st generation biofuels. Lignocellulose biomass consists mainly of polysaccharides and lignin. The polysaccharides contain plant cell wall polysaccharides (cellulose, hemicellulose, pectin) and storage polysaccharides (starch, inulin), of which the former are of particular importance for bioprocess feedstock purposes [1]. One way to convert lignocellulose biomass into value-added compounds, such as biofuels, is its enzymatic hydrolysis followed by fermentation. The most studied organism for the fermentation step is the yeast *Saccharomyces cerevisiae*, since it naturally produces high amounts of ethanol, has long history of industrial use, and is tolerant to growth inhibitors (e.g. acetic acid) which are produced during the pretreatment of the biomass [2, 3].

The sugar composition of the lignocellulose hydrolysate depends on the type of plant raw material. The main sugars in monocots, which are considered the main renewable-energy crop, are the hexose d-glucose and the pentoses d-xylose and l-arabinose [4, 5]. Complete substrate utilization is required for an economically competitive biofuel process, but wild-type *S. cerevisiae* is not able to consume these pentose sugars [6]. To circumvent this issue, numerous studies have investigated metabolic engineering of yeast for the utilization of d-xylose and l-arabinose (Reviewed in Refs. [7, 8]). The utilization of d-xylose has received more attention, as in many biomasses it is present in higher abundance than l-arabinose [8, 9]. Nevertheless, some pectin-rich waste biomasses from the food industry (e.g. orange peels, sugar beet pulp) contain higher amounts of l-arabinose than d-xylose [10]. These biomasses have gained interest as industrial feedstocks due to their low lignin content, which decreases the need for pretreatment [11, 12]. They are also abundantly available at low cost, and their utilization does not interfere with food production [11–13].

Besides pathway engineering, several studies have focused on finding an efficient uptake system for d-xylose and l-arabinose (Reviewed in Ref. [9]). Some of the endogenous sugar transporters of yeast can transport these sugars, but with low affinity and thus the transport is inhibited by d-glucose [9, 14, 15]. Although it has been stated that transport is currently not the limiting factor in either d-xylose or l-arabinose fermentation, a high-affinity pentose transporter could improve growth in conditions where the substrate concentration is low and enable simultaneous utilization of d-glucose and pentoses [8, 9, 16]. Several heterologous pentose transporters have been characterized to investigate their suitability for such purpose [9]. l-Arabinose transporters have thus far been found from yeasts [15, 17, 18], fungi [16, 19, 20], bacteria [21–24] and from the plant *Arabidopsis thaliana* [15, 25]. However, many of these transporters suffer from low expression levels in yeast and from inhibition of l-arabinose transport by d-glucose. [9].

*Trichoderma reesei* is an ascomycete fungus well-known for its ability to secrete high amounts of cellulases and hemicellulases. Due to its saprophytic lifestyle, it can grow on many biomass-derived sugars, including l-arabinose. Although its genome has been predicted to contain roughly 50–100 sugar transporter coding genes [26–28], less than 10 transporters have been functionally characterized [14, 26, 29–33]. To increase our knowledge of *T. reesei* sugar transportome and to identify novel l-arabinose transporters with potential in biotechnological applications, we set out to investigate l-arabinose transporters from *T. reesei*.

We started our investigation by analyzing a transcriptome data set from a previously published study, which investigated the role of *T. reesei* transcription factors XYR1 and ARA1 on gene expression in the presence of l-arabinose and d-galactose. XYR1 is the master regulator of cellulase and hemicellulase expression in *T. reesei* along with another transcription factor, ACE3 [34, 35]. On the other hand, ARA1 has been found to regulate the expression of genes involved in l-arabinose and d-galactose metabolism and, additionally some genes involved in pectin metabolism [36]. XYR1 is also involved in the regulation of l-arabinose metabolism, supported by the fact that deletion of both *xyr1* and *ara1* is required for removal of *T. reesei* growth on l-arabinose [36]. It has been previously observed that the regulatory targets of XYR1 include sugar transporters [34, 37]. Since the genetic and functional orthologs of ARA1 in other fungi regulate the expression of sugar transporters as well, we were interested to see if this is the case also in *T. reesei* [38, 39]. As transporter expression was not analyzed or discussed in the original article about *T. reesei* ARA1 [36], we analyzed the deposited data set to identify sugar transporter genes that were highly expressed on l-arabinose and directly regulated by ARA1, and which thus might play a role in the transport of l-arabinose.

Several known and putative transporters were highly expressed on l-arabinose in an ARA1- or XYR1-dependent manner. To narrow our search, we conducted phylogenetic analysis and investigated the presence of sequence determinants that have been observed to be important regarding pentose utilization [9]. Trire2_104072 was identified as one of the potential l-arabinose transporters. This transporter was previously identified in our institute in 2007 in a study where a *T. reesei* cDNA library was screened for clones that would allow yeast growth on d-xylose, but its kinetics for d-xylose or its ability to transport other sugars were not investigated [14]. Since it was the first d-xylose transporter identified from *T. reesei*, it was named XLT1 (d-**x**y**l**ose **t**ransporter 1). After the discovery of this transporter, several other d-xylose transporters have been identified from *T. reesei* [26, 29, 32]. Thus far, no specific l-arabinose transporters have been characterized from *T. reesei*, although a deletion strain of one of the d-xylose transporters has been reported to have a growth defect on l-arabinose and one d-glucose transporter has been shown to have minor l-arabinose transport activity [29, 40].

Accordingly, we decided to assess Trire2_104072 as a possible l-arabinose transporter. For that end, we chose both yeast and *Xenopus* model systems for the functional characterization of Trire2_104072. Yeast has been used widely for the characterization of transporters from fungal and plant origin, and it was also used in the original study about Trire2_104072 [14]. The electrophysiological two-electrode voltage clamp (TEVC) method [41], which utilizes oocytes from the African clawed frog *Xenopus laevis*, has also been used for the characterization of electrogenic sugar transporters (e.g. sugar/proton symporters). Since its use for the characterization of fungal transporters has been rare, we decided to test its suitability for this purpose. This investigation resulted in interesting findings about Trire2_104072, which could serve as a new tool for improving the pentose-utilizing yeast strains.

## Results

### Trire2_104072 is highly expressed on l-arabinose in ARA1-dependent manner

To identify potential l-arabinose transporters we analyzed a published transcriptome data set for the expression of sugar transporter genes [36]. In the study by Benocci et al., *T. reesei* strain QM9414 and its $\Delta$*xyr1*, $\Delta$*ara1* and $\Delta$*xyr1*$\Delta$*ara1* deletion mutants were cultured on medium containing l-arabinose or d-galactose as the sole carbon source, and subjected to transcriptome analysis [36]. The set of genes we chose for the analysis included characterized and putative transporter genes from *T. reesei* (see “Methods”). Expression levels and log_2_ fold-changes obtained for the chosen transporters are shown in Additional file 1: Figure S1.

From the data it could be seen that several transporters were expressed at high levels on these carbon sources in the parental QM9414 strain (Additional file 1: Figure S1a). When this strain was grown on l-arabinose, the transporter genes with the highest expression levels were the thus far uncharacterized Trire2_82309, d-xylose transporter Trire2_104072 (*xlt1*) [14], d-glucose/d-xylose transporter Trire2_50894 (*str1*) [26, 29] and d-glucose facilitator Trire2_47710 (*stp1*) [30, 42]. Trire2_82309 and Trire2_104072 were downregulated in mutants lacking ARA1, while *str1* was downregulated in mutants lacking XYR1 (Additional file 1: Figure S1b). In contrast to these genes whose expression was downregulated in the absence of ARA1 or XYR1, *stp1* appeared to be upregulated in the double deletion mutant. Other transporter genes that were differentially expressed in the mutant strains on l-arabinose were the cellobiose/lactose transporter Trire2_3405 (*crt1*) [30, 33, 43], which was downregulated in the absence of XYR1, and the d-xylose/d-mannose/cellobiose transporter Trire2_69957 [44], which was regulated by both XYR1 or ARA1 l-arabinose.

Regarding d-galactose, only Trire2_82309 and STP1 were highly expressed on this carbon source (Additional file 1: Figure S1a). Interestingly, STP1 was shown to be able to transport d-galactose and small amounts of l-arabinose in a previous study [40]. As with l-arabinose cultures, Trire2_82309 and Trire2_104072 were downregulated in strains lacking ARA1 (Additional file 1: Figure S1b). Trire2_62380 (*str3*) [26] and Trire2_22912 (*hxt1*) [45] were regulated somewhat similarly on both carbon sources, with the former being upregulated in the absence of ARA1 and the latter in the absence of XYR1 (Additional file 1: Figure S1b)

The fact that Trire2_82309 and Trire2_104072 were highly expressed on l-arabinose and downregulated in the mutant lacking ARA1, which is a positive regulator of genes related to l-arabinose metabolism [36], indicates that they might be involved in l-arabinose metabolism of *T. reesei*. Since Trire2_104072 was highly expressed only on l-arabinose and regulated more strictly by ARA1 on l-arabinose than Trire2_82309, we decided to focus on it in our further analysis.

### Trire2_104072 is homologous to l-arabinose transporter from *Penicillium chrysogenum*

The gene coding for Trire2_104072 is located on the scaffold 3 of the *T. reesei* QM6a genome assembly (position 68870–70589) [46]. The same genomic region contains another putative transporter, Trire2_56684 and a putative transcription factor, Trire2_104075. This region is part of the light-regulated cluster described by Stappler et al. [47], which also contains cellulase genes *cbh2* and *egl2*.

**Fig. 1:**
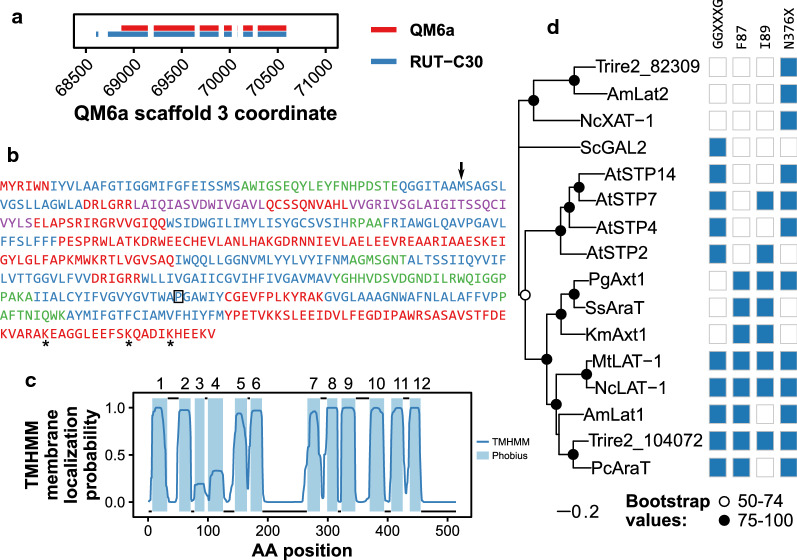
Sequence, localization and phylogenetic analysis of the l-arabinose transporter Trire2_104072. **a** Difference between exons in QM6a and RUT-C30 genome annotations of Trire2_104072 **b** Amino acid sequence of Trire2_104072 colored according to localization predicted by TMHMM (red = intracellular, blue = membrane, purple = membrane with lower probability, green = extracellular). The arrow denotes the start codon at the QM6a genome annotation and the asterisks denote the three predicted medium confidence ubiquitination sites. The proline at position 388 which is mutated in XLT1 is marked with a rectangle **c** Plot of the predicted membrane localization. Line shows the predicted membrane localization probability calculated by TMHMM, while the colored segments show transmembrane domains predicted by Phobius. Numbers of the TMDs are indicated on top of the segments. **d** Maximum-likelihood tree of Trire2_104072 with other published transporters capable of transporting l-arabinose and l-arabitol (AmLat2). The scale bar indicates number of substitutions per site, and the symbols at the internal nodes present bootstrap support values from 100 runs. The presence of sequence determinants discussed in text is indicated by shaded box. Am, *Ambrosiozyma monospora*; Nc, *Neurospora crassa*; Sc, *Saccharomyces cerevisiae*; At, *Arabidopsis thaliana*; Pg, *Pichia guilliermondii*; Ss, *Scheffersomyces stipitis*; Km, *Kluyveromyces marxianus*; Mt, *Myceliophtora thermophila*; Pc, *Penicillium chrysogenum*

A comparison of the sequences of Trire2_104072 from *T. reesei* QM6a and RUT-C30 (TrireRUTC30_33630) genomes revealed differences between the sequence annotations, as we observed in our previous study with another *T. reesei* membrane transporter CRT1 [33, 46, 48]. The nucleotide sequence found from the QM6a genome annotation is shorter. It contains only 7 exons, while the RUT-C30 version contains one extra exon in the N-terminus. Additionally, the second exon is longer as shown in Fig. 1a. Due to its shorter length, the QM6a version of the protein is predicted by TMHMM [49] to not contain the 12 transmembrane domains (TMD) which are characteristic of major facilitator superfamily (MFS) sugar transporters (Fig. 1b). The RUT-C30 version is estimated to contain 12 TMDs when modelled by CCTOP or Phobius [50, 51], but the predicted probabilities for TMDs 3–4 are below 0.5 in the TMHMM prediction (Fig. 1c). The N-terminal intracellular part of the protein is relatively short, while the C-terminal intracellular tail is longer and contains three lysine residues which were predicted to be ubiquitination sites with medium confidence by the UbPred server [52]. The translation of the cDNA sequence (GenBank accession number AY818402) which was used in the previous report about this transporter is identical to the RUT-C30 version except that it contains a P388H mutation at the 11th TMD (Fig. 1b) [14]. From now on the version with the P388H mutation will be referred to as XLT1, whereas the version in the RUT-C30 genome annotation will be referred to as Trire2_104072.

Figure 1d shows a phylogenetic alignment of Trire2_104072 with some other transporters which have been reported to be capable of transporting l-arabinose when expressed in yeast. Trire2_104072 groups together with the recently discovered *Penicillium chrysogenum* l-arabinose transporter AraT (64% identity with BLASTp) [16]. These two transporters, along with the two other l-arabinose transporters from filamentous fungi (*Neurospora crassa* LAT-1 and *Myceliophtora thermophila* LAT-1 [20]) and a transporter from l-arabinose-fermenting yeast (*Ambrosiozyma monospora* Lat1) [18, 53] form their own clade. The three l-arabinose transporters from non-conventional yeasts (*Pichia guilliermondii*, *Scheffersomyces stipitis* and *Kluyveromyces marxianus* [15, 54]) and the four transporters from plant *Arabidopsis thaliana* [25] also form their own clades. While all these transporters fall under the same large monophyletic clade, some transporters cluster under different clades. For example, *A. monospora* Lat2 [18, 53], Trire2_82309 and *N. crassa* XAT-1 [19] group together outside the previously discussed major clade. Although initially published as an l-arabinose transporter [18], Lat2 was later found to be an l-arabitol and ribitol transporter rather than an l-arabinose transporter [53]. Besides Gal2, which forms its own clade in Fig. 1d, two other *S. cerevisiae* transporters have been discovered to be able to transport l-arabinose (Hxt9, Hxt10) [15]. However, since they have been shown to be less efficient at supporting growth on l-arabinose than Gal2 [15], they were excluded from this analysis.

The prediction of substrate specificities of sugar transporters based on their amino acid sequence has proven to be difficult. However, at least four sequence determinants which are believed to be important for pentose transport and d-glucose inhibition have been identified. To complement the phylogenetic analysis, we surveyed the presence of these motifs from transporters shown in Fig. 1d. Of the four sequence determinants, the first three are located in the first TMD and the fourth in the seventh TMD. The first sequence determinant is the G-G/F-X-X-X-G motif (residues 81–86 in Gal2) that was discovered to be involved in the ability to transport d-xylose [55]. The transporters with this motif are shown in Fig. 1d, and this part of the alignment is shown in Additional file 1: Figure S2. The phenylalanine residue (position 85 in Gal2) before the last glycine in this motif is conserved in all of these transporters. Mutagenesis of this residue has been reported to improve l-arabinose transport by Gal2 and d-glucose transport by *T. reesei* STP1, but also to reduce d-glucose and d-xylose transport by *P. guilliermondii* Mgt05196 and d-xylose transport by *Escherichia coli* XylE [40, 42, 56, 57].

The second sequence determinant is the phenylalanine residue (position 87 in Gal2), which follows the last glycine of the first sequence motif [54]. In transporters that prefer d-xylose as the substrate this phenylalanine is replaced by tyrosine [54]. The transporters in Fig. 1d can be grouped into two clades based on this motif alone, and majority of the transporters have either phenylalanine or tyrosine in this position. This residue is followed by aspartate in all transporters except Trire2_104072, which has glutamate in this position. Mutation of this residue from aspartate to alanine was found to abolish d-glucose and d-xylose transport in Trire2_63966 [32]. The third sequence determinant, which has been shown to play a role in specificity for l-arabinose, is the tyrosine at position 89 in Gal2 [58]. Mutation of this residue to isoleucine altered the *K*_m_ of Gal2 for l-arabinose from 335 to 99 mM and lowered the ratio of *K*_m_ values of l-arabinose and d-glucose tenfold from 176 to 15 [58].

The fourth sequence determinant is the asparagine at position 376 in *S. cerevisiae* Gal2 (TMD 7), which has been implicated to be important for d-glucose inhibition [9]. Asparagine is replaced by phenylalanine in all transporters which possess the second motif (F87), except the two which still have asparagine in this position (*S. stipitis* AraT and *K. marxianus* Axt1). Mutation of this residue from asparagine to phenylalanine was found to abolish d-glucose transport and decrease *K*_m_ for d-xylose in Gal2 [59]. Other studies have also shown significance of this residue for different transporters [32, 40, 56]. Besides sequence determinants in TMDs 1 and 7, TMD 6 also contains a motif (YFFYY in Gal2) which has been shown to be important for pentose transport [32, 40, 56, 57, 60]. Interestingly, all of the transporters which possess the second sequence determinant (F87) also have valine at the first position of this motif (Additional file 1: Figure S2). The proline residue which has been mutated to histidine in XLT1 (position 452 at Gal2) is conserved in most of the transporters, and it is not replaced by histidine in any of them. Of these transporters, only *N. crassa* LAT-1, *M. thermophila* LAT-1 and Trire2_104072 possess all of the sequence determinants shown in Fig. 1d. The presence of important sequence determinants and homology to a specific l-arabinose transporter further suggested that Trire2_104072 might function as a l-arabinose transporter.

### Functional analysis of Trire2_104072 with electrophysiological methods

**Fig. 2:**
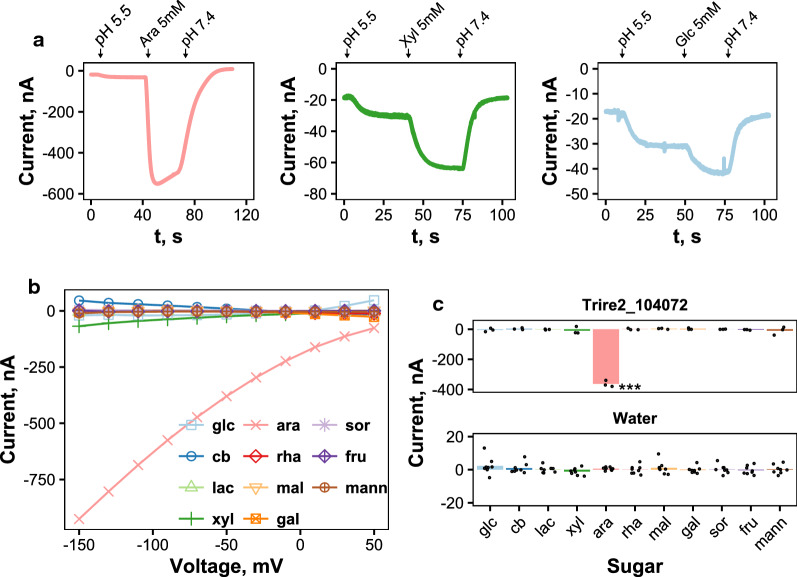
Electrophysiology studies of Trire2_104072. **a** Current traces from a representative TEVC experiment where Trire2_104072-expressing oocyte clamped to − 50 mV was perfused with 5 mM l-arabinose (left), 5 mM d-xylose (middle) or 5 mM d-glucose (right) **b** I–V curves from a TEVC experiment where a Trire2_104072-expressing oocyte was perfused with different sugar solutions with 5 mM concentration and the sugar-induced currents were measured at different clamping voltages. Results from a representative experiment are shown. glc, d-glucose; cb, cellobiose; lac, lactose; xyl, d-xylose; ara, l-arabinose; rha, l-rhamnose; mal, maltose; gal, d-galactose; sor, l-sorbose; fru, d-fructose; mann, d-mannose. **c** Selectivity plot of oocytes injected with water or Trire2_104072 mRNA. Currents induced by different sugars at − 50 mV are shown. Points present results from different oocytes and bars present their average. Significance was estimated with ANOVA and Tukey’s test (***: *p* < 0.005)

We performed functional characterization of Trire2_104072 with the electrophysiological TEVC method which utilizes *X. laevis* oocytes. The gene was ligated into a *X. laevis* expression vector, which allowed us to in vitro transcribe mRNA which was then injected into the oocytes. Since the QM6a version did not possess the 12 TMDs typical of MFS transporters, we used the RUT-C30 version of the gene for the functional analysis. The oocytes injected with Trire2_104072 mRNA were tested for transport activity of different sugars. In contrast to oocytes injected with water as a control (data not shown), oocytes injected with Trire2_104072 mRNA transported d-xylose, as expected, and also l-arabinose and d-glucose, as witnessed by analysis of the current traces (Fig. 2a). The traces also indicated that l-arabinose induces higher currents than d-xylose or d-glucose, suggesting that this sugar is transported with the highest rate. The currents induced by d-glucose were lower than those for l-arabinose and d-xylose. To further study the selectivity of Trire2_104072, we measured current as a function of voltage (I–V curve) in the presence of different sugars (Fig. 2b). Significantly higher negative currents were seen for l-arabinose (about − 350 nA at − 50 mV) as shown in Fig. 2c, but the currents for other sugars, including d-xylose, were small at this voltage. No sugar-induced currents were seen in the I–V curves obtained from water-injected oocytes (Fig. 2c). The presence of sugar-induced currents indicates that the transport was electrogenic, and that Trire2_104072 functions as a sugar/proton symporter.

**Fig. 3:**
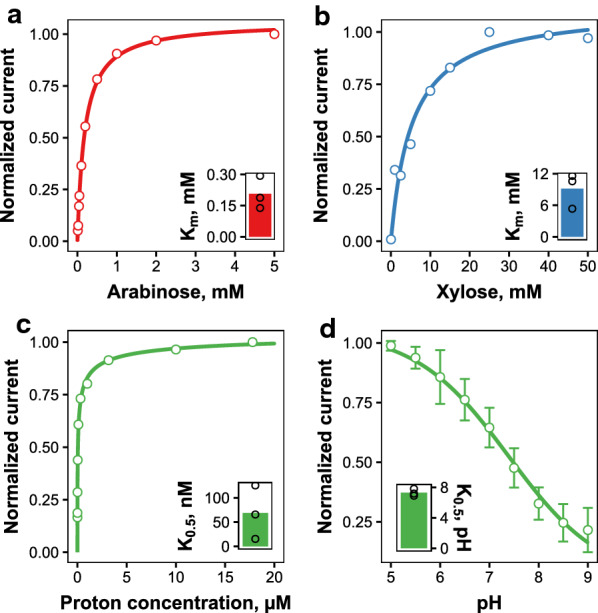
Kinetics determination of Trire2_104072 with electrophysiology. **a**–**c** Representative experiments of the kinetics studies for l-arabinose (**a**), d-xylose (**b**) or proton concentration (**c**) at − 50 mV. Kinetics for proton concentration were determined in the presence of 5 mM l-arabinose. The values were normalized to the lowest current obtained for each sugar at − 50 mV. Insets show the predicted *K*_m_ (**a**,** b**) or *K*_0.5_ (**c**) values (points) and their mean (bar) from three different oocytes. Line shows the Michaelis–Menten (**a**,** b**) or Hill (**c**) kinetics prediction. **d** Normalized current as a function of pH at -50 mV. Points and error bars present mean and standard deviation between currents obtained from three different oocytes, and line shows the Hill kinetics prediction. Inset shows the predicted *K*_0.5_ values as pH, with presentation as in **a**–**c**

Kinetic studies revealed that Trire2_104072 is a high-affinity transporter for l-arabinose with *K*_m_ of $0.207 \pm 0.079$ mM (mean ± standard deviation, *n* = 3) at pH 5.5 and − 50 mV, as shown in Fig. 3a. Regarding d-xylose, it is a low-affinity transporter with *K*_m_ of $9.16 \pm 3.35$ mM in the same conditions (Fig. 3b). The *I*_max_ values for l-arabinose and d-xylose were − $355 \pm 72$ nA and − 72.3 ± 22.3 nA at pH 5.5 and − 50 mV. Current as a function of proton concentration followed Hill kinetics with *K*_0.5_ of $68.9 \pm 55.1$ nM (pH 7.3 ± 0.47) at − 50 mV when determined in the presence of 5 mM l-arabinose (Fig. 3c). This high affinity for protons causes Trire2_104072 to retain about 25% of its maximum transport activity even at pH 9 (Fig. 3d). The affinity of Trire2_104072 for protons is somewhat similar to that observed for *U. maydis* sucrose transporter Srt1 (*K*_0.5_ pH 7.3 for Trire2_104072, *K*_m_ pH 7.7 for Srt1) [61].

Analysis of the kinetics at different voltages revealed that higher transport rates are obtained at lower voltages (Additional file 1: Figure S3a–c). There were no significant differences in the voltage-dependence of *I*_max_ between the substrates (Additional file 1: Figure S3d). Regarding the dependence of *K*_m_ value on voltage, opposite trends were seen for l-arabinose and protons, although variation between the experiments was high (Additional file 1: Figure S3e–f). The Hill coefficient of the pH-dependence increased as the voltage became more depolarized (Additional file 1: Figure S3g). The values obtained for the Hill coefficient were below 1, which indicates negative cooperativity.

The shape of the curves during I–V measurements suggested that this transporter exhibits pre-steady state currents (Additional file 1: Figure S4a). Pre-steady state currents are found only from certain symporters, and they are thought to be caused by the movements of the empty transporter in response to voltage jumps [62, 63]. The charge movements were extracted from the currents and they were found to be inhibited by the presence of l-arabinose (Additional file 1: Figure S4b). To study this phenomenon further, we measured the charge movements at different pH values in the absence of l-arabinose. Charge movements increased with proton concentration until pH 6.5–7, after which they started to decrease again (Additional file 1: Figure S4c–d). The dependence of V_0.5_ and Q_max_ on pH followed a similar trend between the two tested oocytes, although the magnitudes were different (Additional file 1: Figure S4e–f). The maximal charge transfer can be used for the calculation of transporter turnover rate, indicating the number of sugar molecules transported per unit time. The turnover numbers for l-arabinose and d-xylose were $60.2 \pm 16.7$ s^−1^ and $12.3 \pm 2.85$ s^−1^ (mean ± standard deviation, *n* = 3) at pH 5.5. Similar values have been obtained for other transporters, e.g. human SGLT1 (28 s^−1^) and *A. thaliana* STP1 (59 s^−1^) [64, 65].

### Characterization of Trire2_104072 in yeast

**Fig. 4:**
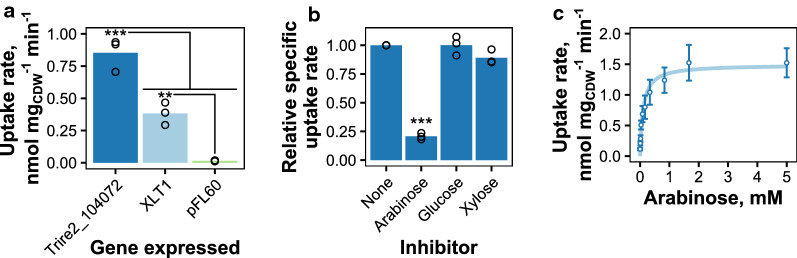
l-arabinose uptake studies with Trire2_104072 expressed in yeast. **a** Comparison of l-arabinose uptake rates of yeast strains transformed with Trire2_104072, XLT1 (Trire2_104072^P388H^) expression plasmids or with the control plasmid. Significance was estimated with ANOVA and Tukey’s test (**: *p* < 0.01, ***: *p* < 0.005). Arabinose concentration was 0.167 mM. Points present results from three individual experiments and bars their average. **b** Inhibition of l-arabinose transport of Trire2_104072. Arabinose concentration was 0.167 mM and the concentration of the inhibiting sugar was 0.833 mM. Presentation and statistics as in panel a. **c** Kinetics of l-arabinose uptake of Trire2_104072 at pH 6.5. Points are average values from three independent experiments and the error bars present the standard deviation between them. Line presents the Michaelis–Menten kinetics prediction

We also performed experiments with Trire2_104072 in yeast to complement the electrophysiological studies. We included XLT1 (Trire2_104072^P388H^) in the analysis, since the mutation of proline to histidine can be considered as a radical replacement, which could affect the transport properties. The ability of the resulting strains to uptake l-arabinose was tested with uptake experiments with ^14^C-labeled l-arabinose. The l-arabinose uptake rate was clearly increased by the expression of Trire2_104072 or XLT1 in comparison with the control strain which was transformed with the empty pFL60 plasmid (Fig. 4a). The strain expressing Trire2_104072 had about twofold higher transport rate than the strain expressing XLT1. To test if the uptake of l-arabinose was inhibited by other sugars, we measured l-arabinose uptake in the presence of other sugars in a competition assay where the competing sugar was present in fivefold excess (Fig. 4b). No inhibition was seen with d-glucose ($0 \pm 8.2$%, mean ± standard deviation), but d-xylose appeared to be slightly inhibitory ($10.9 \pm 6.3$%). As expected, l-arabinose itself inhibited the transport. Similar inhibition percentages were obtained for XLT1 (d-glucose: $4.5 \pm 17.6$%, d-xylose: 13.4 ± 7.9%), which suggests that the P388H mutation did not affect the substrate specificity. Analysis of l-arabinose transport kinetics in yeast revealed somewhat similar results to the *Xenopus* results, as shown in Fig. 4c. *V*_max_ and *K*_m_ values of $1.49 \pm 0.07$ nmol mg_CDW_^−1^ min^−1^ (mean ± std. error of the prediction) and $0.102 \pm 0.020$ mM were obtained at pH 6.5. The *K*_m_ value obtained in yeast is about half of that obtained in oocytes.

**Fig. 5:**
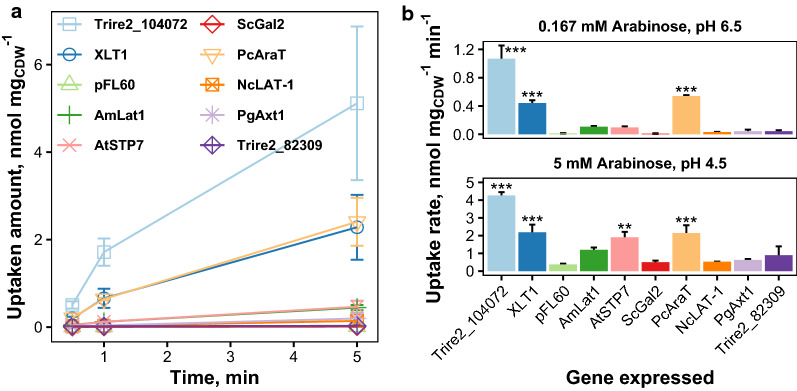
Comparison of l-arabinose uptake by different transporters when expressed in yeast. **a** Amount of l-arabinose uptaken by yeast strains expressing different l-arabinose transporters in assay with 0.167 mM l-arabinose at pH 6.5. Error bars present standard deviation between 2–3 independent experiments. **b** l-arabinose uptake rates at two different conditions: 0.167 mM l-arabinose at pH 6.5 (top) and 5 mM l-arabinose at pH 4.5 (bottom). Error bars in present standard deviation between two independent experiments with two separate transformants. Strains with significantly higher uptake rate than control (pFL60) are indicated. Significance was estimated with ANOVA and Tukey’s test (***: *p* < 0.005, **: *p* < 0.01)

We then compared these two transporters to few other published l-arabinose transporters: *P. chrysogenum* AraT, *N. crassa* LAT-1, *P. guilliermondii* Axt1, *A. thaliana* STP7, Trire2_82309 and *S. cerevisiae* Gal2. All genes were expressed from similar expression vectors (2 µm, *URA3* selection) and with the *PGK1* promoter except for *GAL2*, which was expressed with the *ADH1* promoter. All genes were codon-optimized for yeast expression, except *A. monospora* *LAT1* and *A. thaliana* STP7, for which cDNA sequences were used. Figure 5a shows the amount of l-arabinose uptaken by the yeast cell suspensions as a function of time with 0.167 mM l-arabinose. Yeast strains expressing Trire2_104072, XLT1 and *P. chrysogenum* AraT were able to uptake higher amounts of l-arabinose when compared to the other strains. Since Trire2_104072 has high affinity for both l-arabinose and protons, we also did experiments with higher l-arabinose concentration and lower pH, which also reflect actual fermentation conditions better. Figure 5b shows results from both conditions, and from the figure it can be seen that the strains which expressed Trire2_104072, XLT1 or *P. chrysogenum* AraT had significantly higher l-arabinose uptake rates than the control strain (pFL60) in the both tested conditions (*p* < 0.005). The strain expressing *A. thaliana* STP7 had also significantly higher uptake in the condition with higher l-arabinose concentration and lower pH (*p* < 0.01). This result is in agreement with its previously determined pH optimum of 4 [25]. *A. monospora* Lat1 also had slightly higher uptake rate in this condition, but it was not significantly higher than the uptake rate of the control strain. In both conditions, the strain expressing Trire2_104072 had significantly higher uptake rate (*p* < 0.005) than the other strains which had significant uptake rates in relation to the control strain.

### Trire2_104072 is not essential for growth of *T. reesei* on l-arabinose

Given our findings, we were interested in the role of Trire2_104072 during growth of *T. reesei* on l-arabinose. The arabinolytic system of *T. reesei* is thought to be induced by either l-arabinose or its downstream metabolite arabitol, which is formed intracellularly from l-arabinose via d-xylose reductase XYL1 [66]. We hypothesized that the induction of the arabinolytic system might be disrupted in the absence of Trire2_104072 due to inducer exclusion.

To test our hypothesis, the gene coding for Trire2_104072 was deleted from *T. reesei* strain M44. The deletion was confirmed with PCR, which verified the correct integration of the deletion cassette and the absence of Trire2_104072 open reading frame (data not shown). Additionally, we confirmed the absence of Trire2_104072 expression with RT-qPCR. For this, the parental and deletion strains were first cultivated on minimal medium with sorbitol as the carbon source. Sorbitol can be considered a neutral carbon source for *T. reesei*, as it does neither repress or induce cellulase or hemicellulase production [67]. After sufficient biomass had been reached, either d-glucose or l-arabinose was added to 1% concentration before harvesting the mycelium 6h later. As shown in Additional file 1: Figure S4a, Trire2_104072 expression was highly induced on l-arabinose medium in the parental strain, whereas its expression was hardly detected from the deletion strain. These expression values correspond to a log_2_ fold-change of $8.12 \pm 0.23$ in the parental strain between l-arabinose and d-glucose, and to a log_2_ fold-change of − 8.07 ± 0.83 between the deletion and parental strains on l-arabinose (mean ± standard deviation, $n=2$). To see if regulation by ARA1 was affected, we analyzed the expression of the gene coding for extracellular $\upbeta$-galactosidase, *bga1*, whose expression on l-arabinose is regulated mainly by ARA1 [36]. However, no significant differences were seen in the expression of this gene, as it was induced about 3–4 log_2_ fold in both strains when comparing between l-arabinose and d-glucose (Additional file 1: Figure S5a).

To further study the effects of the deletion, we compared total protein secretion and growth of the deletion and wild-type strains. Despite the absence of Trire2_104072, no differences were seen in protein production on minimal medium with 1% l-arabinose as the carbon source, or on spent grain extract (SGE) medium with 4% lactose as the carbon source (data not shown). Spent grain extract has high arabinoxylan content, and has been used by us as an additional inducer for cellulase and hemicellulase production [68, 69]. When grown on plates, the deletion strain did not have an altered phenotype on minimal medium with 1% l-arabinose (data not shown). In liquid cultures, the parental and deletion strains grew similarly in minimal medium with l-arabinose, d-galactose and d-xylose in 1% concentration (Additional file 1: Figure S5b). Small growth improvement, mostly due to shorter lag time, was seen with 1% d-glucose as the carbon source, but we didn’t investigate this further as the effect was small (Additional file 1: Figure S5b).

Since these typically used sugar concentrations are substantially higher (55.5 mM for hexoses, 66.6 mM for pentoses) than the *K*_m_ value of Trire2_104072 for l-arabinose (0.1–0.2 mM), we repeated the experiment with 0.01% concentration of the same sugars. This concentration corresponds to 0.555 mM for hexoses d-glucose and d-galactose, and to 0.666 mM for pentoses d-xylose and l-arabinose. In this concentration, we witnessed a growth defect for the deletion strain on l-arabinose, while growth on the other carbon sources was similar to that observed at 1% concentration (Additional file 1: Figure S5c). Similar experiments performed at l-arabinose concentrations ranging from 0.01 to 0.2% showed that this growth defect is observed at concentrations at or below 0.05%, which corresponds to 3.33 mM (Additional file 1: Figure S5d). These experiments proved that Trire2_104072 functions as a high-affinity l-arabinose transporter in *T. reesei*, and that it is required for growth only at a narrow concentration range.

## Discussion

Although sugar transporters are structurally conserved, their low sequence similarity does not allow the judgement of substrate specificity based on sequence alone. Transcriptome analyses have proven to be useful for the identification of fungal sugar transporters. Of the previously published transporters discussed in this study, PcAraT, NcLAT-1, PgAXT1 and NcXAT-1 were identified with transcriptomic techniques [16, 19, 54, 70]. Transporter identification has been further facilitated by the identification of several fungal transcription factors which regulate gene expression only on specific carbon sources [71]. ARA1, which regulates l-arabinose and d-galactose metabolism in *T. reesei*, is an example of such a transcription factor [36].

Since XYR1 and multiple other transcription factors in *T. reesei* have been shown to regulate the expression of sugar transporters, we hypothesized that the same would be true for ARA1. Of the four genes (Trire2_104072, Trire2_82309, Trire2_50894/*str1*, Trire2_47710/*stp1*) with the highest expression on l-arabinose, two were downregulated in the $\Delta$*ara1* mutant (Trire2_104072, Trire2_82309) and one in the $\Delta$*xyr1* mutant (*str1*). We noted that these genes were significantly downregulated in similar manner in $\Delta$*ara1* and $\Delta$*xyr1* mutants of QM9414 in a previous study [72]. In this study, gene expression upon growth with corn stover or soybean hulls as the carbon source was analyzed. After 4 h of growth on corn stover, Trire2_104072 and Trire2_82309 were downregulated in $\Delta$*ara1* strain and STR1 in $\Delta$*xyr1* strain (see Additional file 4 in Ref. [72]). Similar results were also obtained with soybean hulls as the carbon source, except that STR1 was also downregulated in the $\Delta$*ara1* mutant. However, the downregulation was less severe in this strain than in the $\Delta$*xyr1* mutant (log_2_ fold-changes of − 7.8 vs − 2.1 when compared between the $\Delta$*xyr1* or $\Delta$*ara1* mutants, respectively, and the parental strain at 4 h timepoint).

Since we were interested in specific l-arabinose transporters, we focused our studies on the two transporters regulated solely by ARA1, as both STR1 and STP1 have been shown to transport d-glucose [26, 30, 42]. STR1 also transports d-xylose, but with low affinity compared to d-glucose [26]. Despite its high affinity for d-glucose, it has been suggested to be involved in d-xylose and l-arabinose transport as its deletion leads to a growth defect on these sugars [26, 29].

In contrast to the other genes that were highly expressed on l-arabinose, the fourth gene (Trire2_47710/*stp1*) appeared to be upregulated in the double deletion mutant. The double deletion mutant failed to grow on l-arabinose [36], and therefore this could potentially be part of the starvation response. Upregulation during starvation has been been observed for STP1 previously in comparison to Avicel-grown cultures, and its homolog HGT-1 from *N. crassa* is also upregulated during starvation [30, 73]. Similar upregulation in the double deletion mutant was observed for another transporter, Trire2_121482/STR2, whose expression was discovered to be highest in starvation conditions in a separate study [26]. However, neither *stp1* or *str2* were upregulated in $\Delta$*ara1* or $\Delta$*xyr1*$\Delta$*ara1* mutants when grown on d-galactose, although these mutants were also unable grow on this carbon source. In this case the metabolism of d-galactose was probably more affected rather than its transport, since STP1 itself has been shown to be able to transport d-galactose and was expressed at high levels [40].

After excluding STR1 and STP1 from our search for specific l-arabinose transporters from *T. reesei*, we were left with Trire2_104072 and Trire2_82309. Trire2_82309 was further excluded based on the phylogenetic and sequence analyses. Our hypothesis on Trire2_104072 was verified to be correct by the TEVC analysis, where we observed l-arabinose transport with high affinity. Both Trire2_104072 and its P388H-mutant XLT1 also functioned as l-arabinose transporters in yeast. Expression of Trire2_104072 resulted in higher uptake rate, but this difference could be explained by other reasons than the P388H mutation (e.g. the lack of codon optimization for XLT1). Regarding l-arabinose uptake kinetics, Trire2_104072 appears to be similar to *P. chrysogenum* AraT, *A. thaliana* STP7 and *P. guilliermondii* Axt1, as shown in Table 1. All of them are high-affinity l-arabinose symporters with *K*_m_ values in range of 0.1–0.3 mM. Interestingly, transporters with even higher affinities have been identified (*A. monospora* Lat1, *A. thaliana* STP4 and STP14). Since the details regarding the used expression systems (strains, expression constructs, fusion tags) and assay conditions (e.g. pH) vary between the published studies, direct comparison of *V*_max_ values is not possible. In one report, the inclusion of fluorescent proteins or adenylate kinase as a fusion partner increased the transport rate 3–20 fold, although in some studies no differences, or a difference in the opposite direction, were seen between tagged and non-tagged transporters [53, 74, 75].

**Table 1:** Kinetic parameters of published l-arabinose transporters. *V*_max_ values are reported as nmol min^−1^ mg^−1^ and *K*_m_ values as mM. H^+^ indicates proton/sugar symport mechanism

Organism	Protein	H^+^	Arabinose		Other sugars			References
			*V*_max_	*K*_m_	Sugar	*V*_max_	*K*_m_	
*A. monospora*	Lat1^a^	n.d.	1.1	0.03				[53]
*A. thaliana*	STP2^b^	x	$0.6 \pm 0.08$	$4.5 \pm 2.2$	Gal^d^		0.05	[15, 104]
	STP4^c^	x	$1895 \pm 142$	$0.033 \pm 0.005$	Glc^d^		0.015	[25, 105]
	STP7^c^	x	$230 \pm 13$	$0.29 \pm 0.03$	Gal		28	[25]
	STP14^c^	x	$220 \pm 17$	$0.018 \pm 0.004$	Gal	68.33	0.529	[25, 106]
*K. marxianus*	Axt1		$57 \pm 6$	$263 \pm 57$	Xyl	$3.8 \pm 0.02$	$27 \pm 3$	[54]
*M. thermophila*	LAT-1^e^	x	$171.5 \pm 5.8$	$29.4 \pm 3.6$				[20]
*N. crassa*	XAT-1	n.d.	$1.1 \pm 0.2$	$61.93 \pm 17.68$	Xyl	$0.9 \pm 0.06$	$18.17 \pm 3.23$	[19]
	LAT-1^e^	x	$1945 \pm 50$	$58.12 \pm 4.06$				[20]
*P. chrysogenum*	AraT	x	$5.3 \pm 0.2$	$0.13 \pm 0.03$				[16]
*P. guilliermondii*	Axt1	x	$18 \pm 0.8$	$0.13 \pm 0.04$	Xyl	$8.7 \pm 0.3$	$65 \pm 8$	[54]
*S. cerevisiae*	Gal2		$341 \pm 7$	$371 \pm 19$	Gal	76	25	[54]
			$75 \pm 5.2$	$335 \pm 21$	Glc	$27.2 \pm 0.9$	$1.5 \pm 0.2$	[16, 59]
			$2.2 \pm 0.26$	$57 \pm 11$	Xyl	$91.3 \pm 3.2$	225.6 ± 15.8	[15, 59]
*S. stipitis*	AraT	n.d.	$0.4 \pm 0.06$	$3.8 \pm 1.7$				[15]
*T. reesei*	104072^f^	x	$1.49 \pm 0.07$	$0.102 \pm 0.020$				This study
	104072^g^	x		0.207 ± 0.079	Xyl^g^		9.16 ± 3.35	This study

*n.d.* not determined

^a^mCherry fusion protein was used

^b^protein contained HA epitope

^c^*V*_max_ as nmol min^−1^ mL^−1^

^d^Expressed in *Schizosaccharomyces pombe*

^e^GFP fusion protein was used

^f^Determined in yeast

^g^Determined in oocytes

Several of the transporters listed in Table 1 were tested for l-arabinose uptake in this study. Uptake was tested in two different conditions, since we were concerned that low substrate concentration and high pH would be favorable for Trire2_104072 due to its high affinity for l-arabinose and protons. However, it outperformed the other transporters in both tested conditions. The lack of l-arabinose transport by Trire2_82309 would support its involvement in pentitol transport, as hypothesized on the basis of the phylogenetic analysis, or in the transport of some other sugar. Based on the kinetics values listed in Table 1, *P. chrysogenum* AraT, *N. crassa* LAT-1 and *P. guilliermondii* Axt1 should have been able to uptake l-arabinose at a higher rate than Trire2_104072, but this was not the case. In the original publication, *N. crassa* LAT-1 was tagged with GFP [20], which might increase the transport rate as mentioned before [53]. The *lat-1* sequence that was expressed in this study was also codon optimized, as opposed to the cDNA sequence used in the original publication [20]. This was also the case for *P. chrysogenum* *araT* and *P. guilliermondii* *AXT1* [16, 54]. The sequences of *A. thaliana* *STP7* and *A. monospora* *LAT1* were identical to those used in the original publications, but *A. thaliana* *STP7* was under the control of different promoter [18, 25]. These results underline that the kinetics values reported from yeast uptake experiments are strongly affected by expression and assay conditions.

Besides the effect of assay and expression conditions on the *V*_max_ value, it has also been reported that pH can affect the *K*_m_ value of transporters [61, 76]. Additionally, the membrane potential is also affected by pH and this in turn can also affect the measurements. Membrane potential cannot be controlled in yeast uptake experiments, but it is well controlled by TEVC in the oocyte electrophysiological studies. Oocytes are clamped at a specific voltage, which is not affected by pH. The downside of the TEVC method is that it can only be used with electrogenic transport proteins, which move net charges, e.g. sugar/proton symporters and ion channels. The sugar/proton symport mechanism allows transport of sugars against their concentration gradient at the expense of energy that is consumed by pumping protons across the cell membrane. This enables the saturation of the intracellular metabolic enzymes with their substrates, even though the extracellular concentration would be low [9]. This can be beneficial in the natural environment of saprophytic fungi, where the concentration of sugars is low, and it could explain why many of the fungal sugar transporters appear to be symporters, while most of the yeast sugar transporters are passive facilitators [45, 77]. Majority of the transporters listed in Table 1 use proton symport as their mechanism and thus they could be studied with electrophysiology.

Some of the transporters listed in Table 1 have higher affinity for some other sugar than l-arabinose, suggesting that l-arabinose is not their primary substrate. Additionally, most of the listed transporters have been reported to be able to transport other sugars than l-arabinose, with the exceptions of *P. chrysogenum* AraT and *M. thermophila* LAT-1 (see references in Table 1). It has been reported that the ability to transport l-arabinose is common among the *A. thaliana* STP sugar transporters: of the 13 functional members of the STP family, all but one had some l-arabinose transport activity [25]. Of the *T. reesei* transporters, Trire2_47710 (STP1) has also been shown to have minor l-arabinose uptake activity, although l-arabinose was only slightly inhibitory to d-glucose transport and thus is expected to be only a low affinity substrate for this transporter [40, 42]. These results indicate that the ability to transport l-arabinose might be widespread among MFS monosaccharide transporters, and kinetic studies are needed to investigate which of the transporters are actually specific for l-arabinose.

Regarding the inhibition characteristics of the transporters, the low inhibition of l-arabinose transport by d-glucose observed for Trire2_104072 is remarkable (Table 2). As mentioned previously, inhibition of l-arabinose and d-xylose transport by d-glucose is a problem with yeast pentose utilization [9]. Although the inhibition characteristics of Gal2 have been improved by mutations of the previously mentioned residue (N376), the mutated variants suffer from 20–30 % inhibition by d-glucose when its present in 2-fold excess [58]. The closest homolog of Trire2_104072 among the characterized l-arabinose transporters, *P. chrysogenum* AraT, is inhibited by both d-glucose and d-xylose although it can transport neither of these sugars [16]. The transporter with the most similar inhibition characteristics to Trire2_104072 is *A. thaliana* STP7, which is inhibited more severely by d-xylose than by d-glucose [25]. Majority of the other listed transporters were strongly inhibited by d-glucose or d-xylose, although the ratios of inhibitor and substrate varied strongly between the studies [15, 20, 54].

**Table 2:** Inhibition characteristics of l-arabinose transporters from competition assays

Organism	Transporter	Ratio	Glucose	Xylose	References
*A. thaliana*	STP7	10	22	58	[25]
*A. thaliana*	STP2^a^	1	n.c.	n.d.	[15]
*K. marxianus*	Axt1	4	83	75	[54]
*M. thermophila*	LAT-1^b^	1	${>}$95	n.d.	[20]
*N. crassa*	LAT-1^b^	1	${>}$95	n.d.	[20]
*P. chrysogenum*	AraT	2	63	22	[16]
*P. guilliermondii*	Axt1	100	83	100	[54]
*S. cerevisiae*	Gal2	2	85	29	[16]
*S. stipitis*	AraT^a^	1	n.c.	n.d.	[15]
*T. reesei*	104072	5	$0 \pm 8.2$	$10.9 \pm 6.3$	This study

Ratio is the concentration of the competing sugar divided by the concentration of l-arabinose. Inhibition is shown as percentages

*n.d.* not determined

^a^n.c. = reported as nearly complete inhibition

^b^Value estimated from graph

Based on its kinetic parameters and inhibition characteristics, Trire2_104072 could be a good candidate for metabolic engineering purposes. However, as shown by Bracher et al. [16] in experiments comparing PcAraT and ScGal2, the expression of a high-affinity, low-capacity transporter as the sole l-arabinose transporter might restrict the growth rate compared to the expression of a low-affinity, high-capacity transporter. On the other hand, processes relying solely on the low-affinity transporters suffer from the slowing down of fermentation when the sugar concentration decreases, and their low specificity causes the pentose sugars to be consumed only after d-glucose is consumed [78]. One solution would be to co-express both high-affinity and low-affinity transporters, but since many high-affinity transporters utilize the sugar/proton symport mechanism, concerns have been raised over the formation of a futile cycle [78]. This is because the symporters allow the accumulation of sugars against their concentration gradient at the expense of ATP, whereas the facilitators merely equilibrate the concentration gradient. Yet, it was observed that a strain expressing Gal2 and PcAraT performed better during anaerobic fermentation than a strain expressing Gal2 alone, and thus these concerns might have been only theoretical [58]. In some situations, the reduction of the ATP yield (e.g. by futile cycles or by the utilization of symporters instead of facilitators) might be beneficial because less ATP would be directed towards the formation of biomass [79]. One must also consider the energetic burden caused by the active transport mechanism itself, particularly in anaerobic conditions. A strain expressing PcAraT as the sole l-arabinose transporter failed to grow in anaerobic conditions, possibly due to not meeting the ATP production rate needed for cellular maintenance [16, 58]. Expressing a symporter with higher transport rate could potentially solve this issue.

Besides potentially serving as a tool for improving l-arabinose fermentation of *S. cerevisiae*, the discovery of a highly specific l-arabinose transporter also sheds light into the l-arabinose metabolism of *T. reesei*. l-arabinose is present in the natural habitat of the fungus as part of cell wall polysaccharides, such as arabinoxylan, arabinogalactan and arabinan, of which the first is present in hemicellulose and the others in pectin [1, 80]. The enzymes which are responsible for their degradation are expressed in response to l-arabinose or its downstream metabolite l-arabitol, [66, 80]. The l-arabinose response is coregulated by XYR1 and ARA1 [36]. Deletion of both regulators is needed for removal of growth on l-arabinose or on d-xylose, while deletion of ARA1 alone abolishes growth on d-galactose [36]. The ARA1 ortholog of *N. crassa* (ARA-1) has been recently identified, and its deletion resulted in completely abolished growth on l-arabinose and d-galactose, while growth on d-xylose was not affected [38]. Downregulation of the l-arabinose transporter LAT-1 was observed in the $\Delta$*ara-1* mutant, and strain with *lat-1* deletion was found to be unable to uptake l-arabinose and to have reduced growth on arabinan [38, 70]. In *T. reesei*, deletion of the previously mentioned d-glucose/d-xylose transporter Trire2_50894 (STR1) was found to severely reduce growth on both d-xylose and l-arabinose and their sugar alcohol derivatives [29], which might indicate that it also has l-arabinose transport activity.

Concerning the importance of Trire2_104072 to *T. reesei*, it appears that it plays a more prominent role at low l-arabinose concentrations as we noticed in our study. At higher concentrations its function can be likely compensated by some other transporter (e.g. STR1). It has been shown that 1 mM l-arabinose is enough to result in induction of xylanase genes in *T. reesei* [66], which is below the limit where we observed the growth defect (3.33 mM). These results indicate that the sugar transportome of *T. reesei* is more fine-tuned than what has been previously thought.

It remains to be studied which transporters are responsible for conferring growth on d-xylose and l-arabinose in the $\Delta$*xyr1* and $\Delta$*ara1* mutants. It could be hypothesized that STR1 is able to compensate for the loss of Trire2_104072 transport activity in the $\Delta$*ara1* mutant, but likely not at low l-arabinose concentrations. Similarly, Trire2_104072 could compensate for the loss of STR1 transport activity in the $\Delta$*xyr1* mutant. However, the genome of *T. reesei* possesses many uncharacterized transporters that might be able to transport d-xylose or l-arabinose.

## Conclusions

In this study, the *T. reesei* d-xylose transporter Trire2_104072 was shown to function as an l-arabinose transporter. Analysis of a published transcriptome data set revealed that Trire2_104072 was highly expressed on medium containing l-arabinose and that its expression was dependent on the presence of transcription factor ARA1, which regulates l-arabinose and d-galactose metabolism in *T. reesei*. Studies in two model systems, *Xenopus* and yeast, revealed that Trire_104072 transports l-arabinose with high affinity and d-xylose with low affinity. It also has a high affinity for protons, which ensures that it can operate in a relatively wide pH range. Its physiological role as a high-affinity transporter was suggested by the results from the deletion mutant, which had growth defect only at low concentrations of l-arabinose. At the time of its original discovery, Trire2_104072 was the first d-xylose transporter discovered from *T. reesei*, and now it becomes the first specific l-arabinose transporter that has been characterized from this species. Remarkably, l-arabinose transport by Trire2_104072 was less inhibited by d-glucose and d-xylose than other known l-arabinose transporters. Therefore, this transporter could be valuable for metabolic engineering of yeast for optimal pentose utilization.

## Methods

### Microbial media, cultivation and chemicals

Yeast strains were cultured in rich YPD medium (1% yeast extract, 2% peptone, 2% d-glucose) or in synthetic complete (SCD-Ura) medium (0.67% yeast nitrogen base without amino acids, 2% d-glucose, SC-Ura drop-out mix) [81]. Both media were supplemented with 2% agar for preparing solid media. Yeast was grown in 30 °C with 230 rpm shaking in 50 mL volume in 250 mL flasks.

For growth and protein production studies, *T. reesei* was cultivated on minimal medium containing 15 g/L KH_2_PO_4_, 5 g/L (NH_4_)_2_SO_4_, 1 mL/L trace element stock (FeSO_4_$\cdot$7H_2_O 5 g/L, MnSO_4_$\cdot$H_2_O 1.6 g/L, ZnSO_4_$\cdot$7H_2_O 1.4 g/L, CoCl_2_$\cdot$6H_2_O 3.7 g/L) with indicated carbon source. SGE-lactose medium is minimal medium supplemented with 40 g/L lactose, 20 g/L spent grain extract, 100 mM PIPPS and 8.6 g/L di-ammonium citrate, and with ammonium sulfate replaced with 5.4 g/L Na_2_SO_4_. Both media were adjusted to pH 4.8, and after autoclaving MgSO_4_ and CaCl_2_ were added to 2.4 mM and 4.1 mM final concentrations, respectively. Solid media was supplemented with 20 g/L agar and Triton X-100 (when appropriate) to restrict the spread of the mycelium. For the preparation of spore suspensions, *T. reesei* was grown on potato dextrose agar plates for 3–5 days, and the spores were collected into a solution containing 0.8% NaCl, 0.025% Tween 20 and 20% glycerol and the resulting suspensions were stored in − 80 °C.

24-well plate cultivations were used for the protein production and gene expression studies. Spores were inoculated to 2$\cdot$10^5^ cfu/mL concentration in 4 mL of medium per well, and the plates were incubated in 28 °C with 800 rpm shaking in Infors HT microtron incubator with 3 mm throw (Bottmingen, Switzerland). Growth profiling was done with Bioscreen C incubator (Oy Growth Curves Ab Ltd, Helsinki, Finland) with 200 µL medium per well, and with the same spore concentration as in the 24-well plate studies. Incubation was done in 28 °C with continuous shaking (normal speed, maximal amplitude). Three and five replicate wells per strain were used in the 24-well plate cultivations and in the Bioscreen cultivations, respectively.

Chemicals were obtained from Merck KGaA (Darmstadt, Germany) and molecular biology reagents from Thermo Fisher Scientific (Waltham, MA, USA) unless otherwise mentioned. l-arabinose was obtained from Merck (Darmstadt, Germany), lactose was obtained from VWR (Helsinki, Finland), and l-rhamnose and l-sorbose were obtained from Fluka (Charlotte, NC, USA).

### Molecular biology methods

High-fidelity PCR was done with KAPA HiFi polymerase (Roche, Basel, Switzerland) and colony PCR with DreamTaq polymerase (yeast, *E. coli*) or with Phire polymerase (*T. reesei*). Restriction digestions were performed with restriction enzymes from Thermo Fisher Scientific or NEB (Ipswich, MA, USA). *E. coli* transformations were done with electroporation, yeast transformations with the LiAc/ssDNA/PEG method described by Gietz and Woods [82] and *T. reesei* transformations as described by Penttilä *et al.* [83]. *In vitro* transcription was done with mMessage mMachine T7 kit. Primers were obtained from Thermo Fisher Scientific and they are listed in Additional file 1: Table S1.

### Plasmid and strain construction

The genes coding for Trire2_104072 and Trire2_82309 were ordered as synthetic genes with codon-optimization for heterologous expression in yeast (GeneArt, Thermo Fisher Scientific). For Trire2_104072 we used the version from the RUT-C30 genome assembly (TrireRUT-C30_33630; GenBank ETS04871.1). For Trire2_82309 (TrireRUT-C30_26932, GenBank ETR97140) the versions in the QM6a and RUT-C30 genome assemblies are identical. The yeast expression plasmids for Trire2_104072 and Trire2_82309 were constructed with the Yeast MoClo toolkit [84]. The plasmids harboring the genes in a pMA-T backbone, as well as MoClo plasmids pYTK-2, -11, -51, -67, -74, -82 and -84 were digested with *Bsa*I-HF®v2 and ligated with T4 ligase (both from NEB). The reaction was performed simultaneously in a thermocycler as described by Lee et al. [84]. The resulting plasmids, B11037 and B11554 (Table 3), were sequenced with primers SaSS-19 and -20.

**Table 3:** Plasmids used in this study

Name	Description	References
Electrophysiology		
Pol1	Oocyte expression vector	[107]
B10213	Pol1 with modified multiple cloning site	This study
B10219	B10213 with Trire2_104072	This study
Yeast studies		
B1687	*xlt1* (Trire2_104072^P388H^) in pAJ401 (P_*PGK1*_, *URA3*, 2 µm)	[14]
B2013	Yeast expression vector pFL60 (P_*PGK1*_, *URA3*, 2 µm)	[85]
B2251	*A. monosporaLAT1* in modified pFL60	[18]
B2253	*GAL2* in pVT100-U (P_*ADH1*_, *URA3*, 2 µm)	[86]
B10862	*A. thalianaSTP7* in pFL60	Peter Richard, unpublished
B11037	Trire2_104072 in MoClo-based expression vector (P_*PGK1*_, *URA3*, 2 µm)	This study
B11394	*P. chrysogenumaraT* in pFL60	This study
B11395	*N. crassalat-1* in pFL60	This study
B11396	*P. guilliermondiiAXT1* in pFL60	This study
B11554	Trire2_82309 in MoClo-based expression vector (P_*PGK1*_, *URA3*, 2 µm)	This study
*T. reesei *studies		
B11697	Trire2_104072 deletion vector (*pyr4*)	This study

Genes coding for *P. chrysogenum* AraT (Pc20g01790, GenBank CAP85508.1), *P. guilliermondii* Axt1 (GenBank GZ791040.1) and *N. crassa* LAT-1 (NCU02188, GenBank XP_959582.3) were ordered as synthetic genes with codon optimization for yeast expression (Twist Biosciences, San Francisco, CA, USA). The genes were ligated to pFL60 via Gibson assembly (NEB) and the resulting plasmids (B11394, B11395 and B11396) were verified by sequencing with primers SaSS-98 and -99.

Plasmids B1687, B2013 (pFL60), B2251, B2253 and B10862 were from our institution’s strain collection. pFL60 contains *PGK1* promoter and terminator, *URA3* gene for selection in yeast and the 2 µm replication origin [85]. B1687 contains the *xlt1* gene in pAJ401 backbone which similarly to pFL60 harbors *PGK1* promoter, *URA3* and 2 µm origin [14]. B2251 contains the *A. monospora* *LAT1* gene in modified pFL60 vector [18]. B10862 contains the *A. thaliana* *STP7* gene in pFL60 and was constructed by Dr. Peter Richard of VTT from a plasmid containing *STP7* cDNA which was provided by Dr. Ruth Stadler (University Erlangen-Nürnberg, Erlang, Germany). B2253 contains the *GAL2* gene under the control of *ADH1* promoter in pVT100-U vector [86]. Yeast expression plasmids were transformed into yeast strain H2805 (CEN.PK2-1D: MAT$\upalpha$* ura3-52* *his3*-$\Delta$ *1* *leu2-3,112* *trp1-289* *MAL2-8*^c^ *SUC2*) and transformants were selected on synthetic complete medium which lacked uracil.

For expression in *X. laevis* oocytes, the oocyte expression vector Pol1 was modified to be compatible with the yeast MoClo system. To modify the MCS, the Pol1 plasmid was digested with *Eco*RI and *Bam*HI before ligation of the fragment containing the *Bsa*I sites, which was digested with the same enzymes. The fragment was constructed by annealing oligos SaSS-43 and -44. The MoClo-compatible version of Pol1, which was named B10213, was digested with *Bsa*I and the two resulting fragments (Pol1 contains one endogenous *Bsa*I site) were gel purified. The backbone fragments were ligated with the Trire2_104072 insert, which was liberated from the pMA-T backbone with *Bsa*I. The resulting plasmid B10219 was sequenced with T7 promoter primer and SaSS-57 primer.

Deletion vector for Trire2_104072 was constructed by amplifying the 5′ and 3′ flanking regions from *T. reesei* genomic DNA with primers SaSS-101, -104, -155 and -156. The genomic DNA was prepared with the Easy DNA kit (ThermoFisher Scientific) according to kit instructions. The deletion vector was constructed with yeast homologous recombination using yeast strain FY834 [87, 88]. To do this, the amplicons were transformed to yeast along with pRS426 backbone [89], which was digested with *Eco*RI and *Xba*I and gel purified, and with *pyr4* fragment which was prepared as described by Landowski *et al.* [69]. The resulting transformants were pooled and the plasmids were rescued with glass bead extraction followed by plasmid miniprep and transformed into *E. coli*. Plasmids were extracted from the transformant colonies and verified by digestion and with sequencing with primers T27, T60, T552 and T553. Plasmid harboring the correct sequence (B11697) was transformed into *T. reesei* production strain M44 [69]. The transformant colonies were screened with primers T27, T60, SaSS-166, SaSS-167, SaSS-168 and SaSS-169 to verify correct integration to the Trire2_104072 locus. The correct colonies were purified into homokaryons and screened again with PCR. Spore suspensions created from two different transformants were used for the studies, along with two separate spore suspensions made from M44 which served as biological replicates.

### Preparation of mRNA and *X. laevis* oocyte injection

The pol1-based plasmid containing the Trire2_104072 CDS was linearized with *Nhe*I and in vitro transcribed into mRNA. After the transcription the product was purified with LiCl precipitation according to the kit instructions.

Oocytes were obtained via surgery as described by Clemencon et al. [90]. Briefly, adult female *X. laevis* frogs were anaesthetized with 3-aminobenzoate methanesulfonate (1 g/L in ice-water slurry) and the oocytes were removed with surgery. Oocytes were suspended in modified Barth’s medium with Ca^2+^ (MBM+Ca^2+^; 88 mM NaCl, 1 mM KCl, 2.4 mM NaHCO_3_, 1.57 mM MgSO_4_$\cdot$7 H_2_O, 0.66 mM NaNO_3_, 0.75 mM CaCl_2_, 10 mM HEPES), which was supplemented with penicillin and streptomycin, and the oocyte sacs were separated to 3-4 mm pieces with forceps.

The oocytes were defolliculated by collagenase treatment. The oocytes were washed with MBM-Ca^2+^ (MBM+Ca^2+^ without CaCl_2_), after which they were suspended to the same buffer containing 4 g/L collagenase (NB4, SERVA Electrophoresis GmbH, Heidelberg, Germany) and incubated in rocking shaker for 1 h. After the incubation, the washing and collagenase treatment steps were repeated. Finally the oocytes were washed with MBM+Ca^2+^ and healthy stage V–VI oocytes were picked.

The oocytes were injected with 30 ng Trire2_104072 mRNA in 50 nL volume or with the same volume of water. The injection was done with Nanoject II microinjector (Drummond Scientific, Broomall, PA, USA). After injection the oocytes were suspended in MBM+Ca^2+^ and incubated in 17 °C for 3–7 days.

### Electrophysiology experiments with *X. laevis* oocytes

Two-electrode voltage clamp (TEVC) technique was used for the electrophysiological characterization. In this method, the membrane potential of the oocyte is clamped to a specific voltage and the transport is measured by recording the substrate-induced changes in the membrane current [41]. The measurements were done on a TEVC setup consisting of 2-channel perfusion system, OC-725C Oocyte clamp amplifier (Warner Instruments, Hamden, CT, USA) and Axon Digidata 1440A digitizer (Molecular Devices, San Jose, CA, USA). The system was calibrated with oocyte model cell. Microelectrodes filled with 3 M KCl and with resistance between 0.5–5 M$\Omega$ were used. pClamp software suite (Molecular Devices) was used for the analysis. ND-96 buffer without sodium (100 mM choline, 2 mM KCl, 1 mM CaCl_2_$\cdot$2H_2_O, 1 mM MgCl_2_$\cdot$6H_2_O, 3 mM HEPES) was used for the experiments. Each experiment was done with at least three oocytes derived from at least two different frogs, except for the pre-steady state kinetics which were done with two oocytes derived from two different frogs.

For the current trace recordings, the oocytes were perfused with ND-96 (pH 7.4) and clamped at −50 mV. For each sugar, the oocyte was first perfused with ND-96 (pH 7.4) until the current stabilized, then with ND-96 (pH 5.5) until the current stabilized and finally with ND-96 (pH 5.5) containing 5 mM sugar. Afterwards the current was recovered to baseline by perfusing with ND-96 (pH 7.4). The resulting current traces were recorded with Axoscope program of the pClamp software suite.

The I-V curves were recorded with Clampex program of the pClamp software suite. The oocytes were clamped at − 50 mV, perfused with the test solution until the current stabilized and then 200 ms step-wise changes of membrane potential from − 150 to 50 mV were applied. For each measurement, a recording was done first with buffer alone and then with the same buffer which contained the sugar to be analyzed. The steady-state currents obtained in the absence of sugar were subtracted from the currents obtained in the presence of sugar for each of the tested voltages. Between measurements the oocyte was stabilized by perfusing with pH 7.4 buffer.

The selectivity was measured by recording I–V curves with 5 mM sugar solutions in pH 5.5. For the kinetics experiments I–V curve recordings were done with different sugar concentrations with pH 5.5 buffer. For the pH-dependence experiments, I–V curve recordings were done with ND-96 (with 5 mM l-arabinose) adjusted to different pH values. Normalized currents were calculated by dividing the obtained currents with the highest negative current obtained at − 50 mV during the experiment. The kinetics parameters for l-arabinose and d-xylose were calculated by fitting the normalized currents as a function of substrate concentration to Michaelis–Menten (Eq. 1), where $I_{max}$ = maximum current, [*S*] = substrate concentration and $K_m$ = Michaelis constant. For the calculation of the turnover number (see below), unnormalized currents were used for the fitting to obtain the $I_{max}$. The fitting was done with the nls package for R with the default Gauss–Newton algorithm. The *K*_m_ and *I*_max_ values are presented as mean ± standard deviation from three different oocytes.

1 $$\begin{aligned} I = \frac{I_{max} [S]}{[S] + K_m} \end{aligned}$$

The kinetics parameters for pH-dependency were calculated similarly, but the data was fitted to Hill equation (Eq. 2), where *n* is the Hill coefficient and $K_{0.5}$ is the proton concentration where *I* reaches half of the maximum current $I_{max}$.

2 $$\begin{aligned} I = \frac{I_{max} [S]^n}{[S]^n + K_{0.5}^n} \end{aligned}$$

Pre-steady state kinetics were analyzed from I–V curves which were obtained in ND-96 buffer with different pH values but without sugar. The pre-steady state currents were isolated as previously described [63]. The pre-steady state currents were integrated with respect to time to obtain the charge transfer (Q) which were fitted to the following Boltzmann relation (Eq. 3), where *Q* = total charge, $Q_{hyp}$ = charge transfer at hyperpolarizing limit, $Q_{dep}$ = charge transfer at depolarizing limit, $Q_{max}$ = $Q_{dep} - Q_{hyp}$, *z* = apparent valence, *V* = voltage, $V_{0.5}$ = voltage at the midpoint of charge transfer, *F* = Faraday’s constant, *R* = Gas constant and *T* = temperature. The transporter turnover number is defined as $I_{max}/Q_{max}$ [63], where $I_{max}$ is the maximum current obtained from the Michaelis–Menten fit for the kinetics measurements and $Q_{max}$ value is obtained from I–V curve measurement at pH 5.5 without sugar.

3 $$\begin{aligned} \frac{Q-Q_{hyp}}{Q_{max}} = \frac{1}{1 + \mathrm {exp}\left[ \frac{zF\left( V-V_{0.5}\right) }{RT} \right] } \end{aligned}$$

### Sugar uptake assays

Radioactive ^14^C-1-l-arabinose (MC 2019, lot 165-155-054-A-20020115-SB) from Moravek Biochemicals (Brea, CA, USA) was used for transport experiments. Zero-trans uptake assays were performed as previously described [33]. Briefly, yeast cells were harvested in the exponential phase, washed with ice-cold water and resuspended in ice-cold assay buffer to yield a cell suspension with 40–60 OD/mL. Assay buffer was 100 mM potassium phosphate buffer (pH 6.5 in 20 °C), except in the experiment with lower pH where 100 mM sodium citrate buffer (pH 4.5 at 20 °C) was used. Aliquots of the cell suspension and sugar solutions were kept for 5 minutes in 28 °C water bath before mixing 40 µL cells with 20 µL 3x sugar solution in a glass tube with conical bottom. After incubation of desired length, the reaction was quenched with 10 mL of ice-cold water. The mixture was filtrated with prewetted Whatman GF/C filter paper (GE Healthcare, Helsinki, Finland) and the tube was washed with another 10 mL ice-cold water. The filter paper was transferred into scintillation vial containing 4 mL Ultima Gold XR liquid scintillation cocktail (PerkinElmer, Waltham, MA, USA) and counted with TriCarb 2810 TR scintillation counter (PerkinElmer). Blanks were performed to measure unspecific binding of the sugar to cell surface: 20 $\upmu$L 3x sugar solution and 40 $\upmu$L cells were pipetted into 10 mL ice-cold water, which was treated afterwards in similar way as normal samples. To ensure that the uptake was in the linear range, the uptake rate obtained from two different timepoints was compared.

Uptake rate was calculated by dividing the blank-subtracted counts by reaction time (min), amount of yeast (OD) used and specific activity of the radioactive stock solution (cpm/nmol). The relationship between OD and CDW was measured to be 0.252 mg_CDW_ OD^−1^. Two to three technical replicates were used for each sample. Specific uptake rate was defined as uptake rate subtracted with uptake rate obtained for the control strain with the empty plasmid. Kinetics results were fitted to Michaelis–Menten equation with nonlinear least squares estimation with the R package nls.

### RNA extraction and RT-qPCR

RNeasy plant mini kit (Qiagen, Hilden, Germany) was used for the extraction of fungal total RNA. Cultures of three replicate wells were pooled and subjected to RNA extraction which was done as previously described [33]. Afterwards, 1 µg RNA was treated with DNAse I and reverse-transcribed with Transcriptor First Strand cDNA synthesis kit (Roche) with anchored oligo-dT primers. qPCR was done with Sybr Green I mix (Roche) with Trire2_104072 and *bga1* as the target genes and *Sar1* and *gpd1* as the reference genes. The use of *sar1* for this purpose has been suggested previously [91]. Primers are listed in Additional file 1: Table S1.

The results were analyzed with the $$\Delta$$$$\Delta$$C_p_ method by Livak and Schmittgen [92]. Fold-changes were calculated according to Eq. 4, where $$\Delta C_p$$ is the difference between $$C_p$$ values obtained for the target gene and the reference genes ($$C_{p,target} - C_{p,control}$$). Arithmetic mean of the $$C_p$$ values obtained for the reference genes was used. The term $$2^{-\Delta C_p}$$ was defined as the expression level. The obtained fold-changes were further log_2_-transformed.

4 $$\begin{aligned} \frac{2^{-\Delta C_{p,ara}}}{2^{-\Delta C_{p,glc}}} \end{aligned}$$

### Expression data analysis

Expression data was derived from Gene Expression omnibus (GEO) database (Accession number GSE104606) [93], and it is related to results published by Benocci et al. [36]. In the article, *T. reesei* QM9414 and its $\Delta$*xyr1*, $\Delta$*ara1* and $\Delta$*xyr1*$\Delta$*ara1* mutants were inoculated as spores to complex medium with 2% d-fructose as the carbon source. After 20 h of growth (28 °C, 250 rpm), mycelia were washed and transferred to minimal medium with 25 mM l-arabinose or 25 mM d-galactose as the carbon source. RNA was extracted from mycelium samples for RNA-sequencing 2 h after incubation in minimal medium. RNA-sequencing was performed with Illumina technology [36].

*T. reesei* sugar transporters which have been published were chosen for the analysis. Few putative sugar transporters that have shown interesting properties in previous studies were also included. The published sugar transporters are putative d-glucose transporter Trire2_22912 (HXT1) [45], d-xylose facilitator Trire2_63966 (XLTR1) [32], d-glucose/d-xylose transporters Trire2_50894 (STR1) [26, 29], Trire2_121482 (STR2) [26] and Trire2_62380 (STR3) [26], d-glucose/cellobiose facilitator Trire2_47710 (STP1) [30, 42], d-galacturonic acid transporters Trire2_69026 and Trire2_106330 [11], putative lactose transporters Trire2_79202 and Trire2_77517 [94], cellobiose transporter Trire2_67752 [31], d-xylose/cellobiose/d-mannose transporter Trire2_69957 [44] and cellobiose/lactose transporter Trire2_3405 (CRT1) [30, 33, 43]. The putative sugar transporters were Trire2_106556 which is the homolog of *N. crassa* low-affinity d-glucose transporter GLT-1 [73], Trire2_82309 which has been shown to be upregulated in cellulase-inducing conditions [95], Trire2_56684 which has been shown to be part of light-regulated genome cluster which also contains cellulases *cbh2* and *egl2* and whose promoter has been shown to bind XYR1 [37, 47], and Trire2_46819 which is the closest homolog (72% identity with BLASTp) of the *N. crassa* cellobionic acid transporter CLP-1/CBT-1 in *T. reesei* [96, 97]. Trire2_56684 and Trire2_46819 were also reported to be regulated by transcription factor AZF1 in a recent report [98]. However, Trire2_63966 was omitted from the figures due to its low expression level in all conditions.

### Phylogenetic analysis

The amino acid sequences of the transporters were obtained from GenBank (accession numbers are listed in Additional file 1: Table S2). Sequence alignment was done with MUSCLE [99] and the phylogenetic analysis was performed with maximum likelihood method with 100 bootstrap runs [100]. Both steps were performed with MEGA X software [101]. The tree was visualized with ape package for R [102]. Visualization of the alignment was done with msa package for R using MUSCLE method [103].

## Supplementary Information

**Additional file 1: Table S1.** List of primers used in this study. **Table S2.** Accession numbers for sequences used in the phylogenetic analysis. **Figure S1.** Gene expression analysis of selected* T. reesei* transporters on l-arabinose and d-galactose. **Figure S2.** Subset of the multiple alignment of transporter amino acid sequences. **Figure S3.** Analysis of voltage-dependence of Trire2_104072 kinetics. **Figure S4.** Analysis of pre-steady state kinetics of Trire2_104072. **Figure S5.** Characterization of the ΔTrire2_104072 mutant.

## Data Availability

The datasets generated during the current study are available from the corresponding author on reasonable request.
