# Supplementary material for: Functional characterization of a highly specific l-arabinose transporter from Trichoderma reesei
Source: Microb Cell Fact. 2021 Sep 8;20:177. doi: 10.1186/s12934-021-01666-4 (PMC8425032; doi:10.1186/s12934-021-01666-4)
Supplement: Supplementary file 1 — Additional file 1: Table S1. List of primers used in this study. Table S2. Accession numbers for sequences used in the phylogenetic analysis. Figure S1. Gene expression analysis of selected T. reesei transporters on l-arabinose and d-galactose. Figure S2. Subset of the multiple alignment of transporter amino acid sequences. Figure S3. Analysis of voltage-dependence of Trire2_104072 kinetics. Figure S4. Analysis of pre-steady state kinetics of Trire2_104072. Figure S5. Characterization of the ΔTrire2_104072 mutant. [file 12934_2021_1666_MOESM1_ESM.pdf]

Functional characterization of a highly specific l-arabinose transporter from *Trichoderma reesei*

Supporting information

Sami Havukainen, Jonai Pujol-Giménez, Mari Valkonen, Matthias A. Hediger and Christopher P. Landowski

List of supplementary tables

S1 List of primers used in this study

S2 Accession numbers for sequences used in the phylogenetic analysis

List of supplementary figures

S1 Gene expression analysis of selected *T. reesei* transporters on L-arabinose and D-galactose

S2 Subset of the multiple alignment of transporter amino acid sequences

S3 Analysis of voltage-dependence of Trire2_104072 kinetics

S4 Analysis of pre-steady state kinetics of Trire2_104072

S5 Characterization of the ΔTrire2_104072 mutant

Table S1. List of primers used in this study

Name	Sequence
SaSS-19	GGTCAATGCAAGAAATACATATTTGGTCT
SaSS-20	CATTCGTGACTATAAAATGAATAAACTAAACTATTC
SaSS-43	GATCCGGTATGAGAGACCTGTAAGAGCTGGTCTCAATCCG
SaSS-44	AATTCGGATTGAGACCAGCTCTTACAGGTCTCTCATACCG
SaSS-57	ATTCGGGTGTTCTTGAGGCTGG
SaSS-98	ACAAATATAAAAACCAAAAGATCTACGTACTCGAG
SaSS-99	AGAAAAGAAAAAAATTGATCTATCGGAATTC
SaSS-101	GTAACGCCAGGGTTTTCCCAGTCACGACGGTTTAAACTTATGCGTACCAATGACGG
SaSS-104	GCGGATAACAATTTCACACAGGAAACAGCGTTTAAACAAATCAGAGCGAAACACC
SaSS-155	CCTCAGCCTCTCTCAGCCTCATCAGCCGCCTTGGCGGCGTTCTTTTGC
SaSS-156	AGAGCAGAGCAGCAGTAGTCGATGCTAGGCTTTCACCTCATCTGGGAGAC
SaSS-166	CAACTCTTCCCATGCCTAGC
SaSS-167	ACTGGATTGACGGTGTCACC
SaSS-168	ATCGGATTTGGAACATATATGG
SaSS-169	TTAGACCTTTTCTTCGTGC
T27	TGCGTCGCCGTCTCGCTCCT
T60	TGACGTACCAGTTGGGATGA
T552	CGCTATTACGCCAGCTGG
T553	TTACACTTTATGCTTCCGGCT
104072_fwd[1]	GCAATTGGGCTTTCAACCTCG
104072_rev[1]	CATGCGGGAATATCTCCTTCG
bga1_fwd[1]	CATATTCAGCCTGGAGCCCT
bga1_rev[1]	TGAGCCACATAGTTGTCCGT
sar1_fwd[1]	TCTCCACCCTACTTCTGAG
sar1_rev[1]	CTTGTTGCCCAGGATGAC
gpd1_fwd[1]	TGTCCATTCGTGTCCCTACC
gpd1_rev[1]	GTAGGCCAAGATTCCCTTGAG

[1]Primers used in the RT-qPCR analysis

Table S2. Accession numbers for sequences used in phylogenetic analysis

Organism	Transporter	Accession number
A. monospora	Lat1	AY923868.1
A. monospora	Lat2	AY923869.1
A. thaliana	STP2	NP_172214.5
A. thaliana	STP4	NP_001326818.1
A. thaliana	STP7	NP_001329423.1
A. thaliana	STP14	NP_001185417.1
K. marxianus	Axt1	GZ791039.1
M. thermophila	LAT-1	XP_003663698.1
N. crassa	LAT-1	XP_959582.3
N. crassa	XAT-1	XP_961039.2
P. chrysogenum	AraT	CAP85508.1
P. guilliermondii	Axt1	GZ791040.1
S. cerevisiae	Gal2	NP_013182.1
S. stipitis	AraT	XP_001382755.2
T. reesei	Trire2_104072	ETS04871.1
T. reesei	Trire2_82309	XP_006969603.1

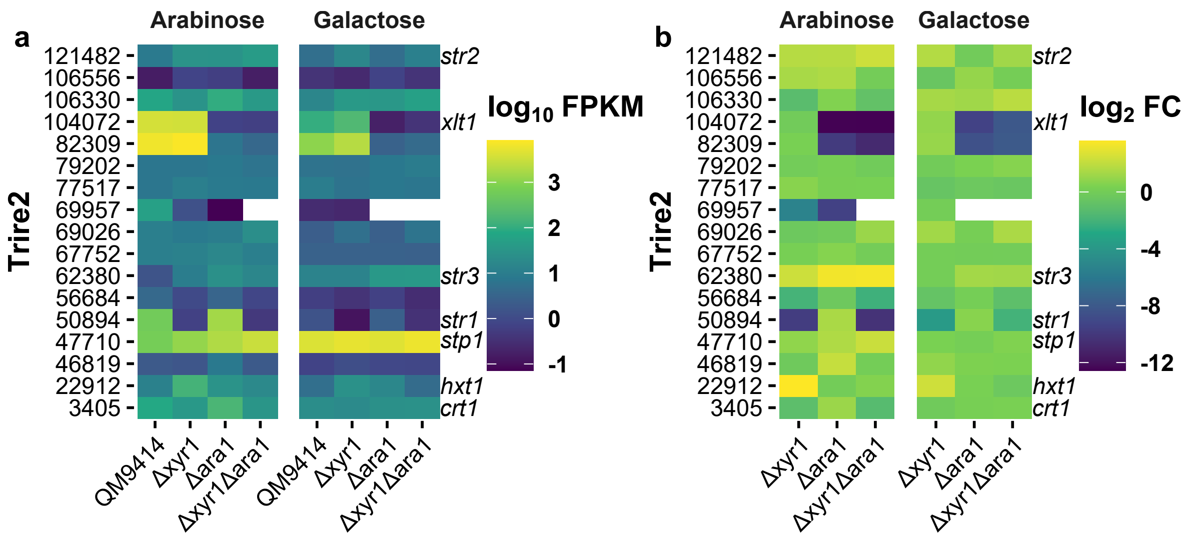

Figure S1. Gene expression analysis of selected *T. reesei* transporters on L-arabinose and D-galactose. **a** Average expression levels (n=2) of selected transporters from data set published by Benocci *et al.* (1). Values are represented as $\log_{10}$-transformed FPKM values to present whole range of expression levels. No Trire2_69957 expression was detected for Δ*xyr1*Δ*ara1* strain on L-arabinose and for Δ*ara1* and Δ*xyr1*Δ*ara1* strains on D-galactose (indicated by white tiles). **b** Average $\log_2$ fold-change values (n=2) between mutant strains and QM9414. Trire2 protein IDs and previously given names are shown next to the heatmaps.

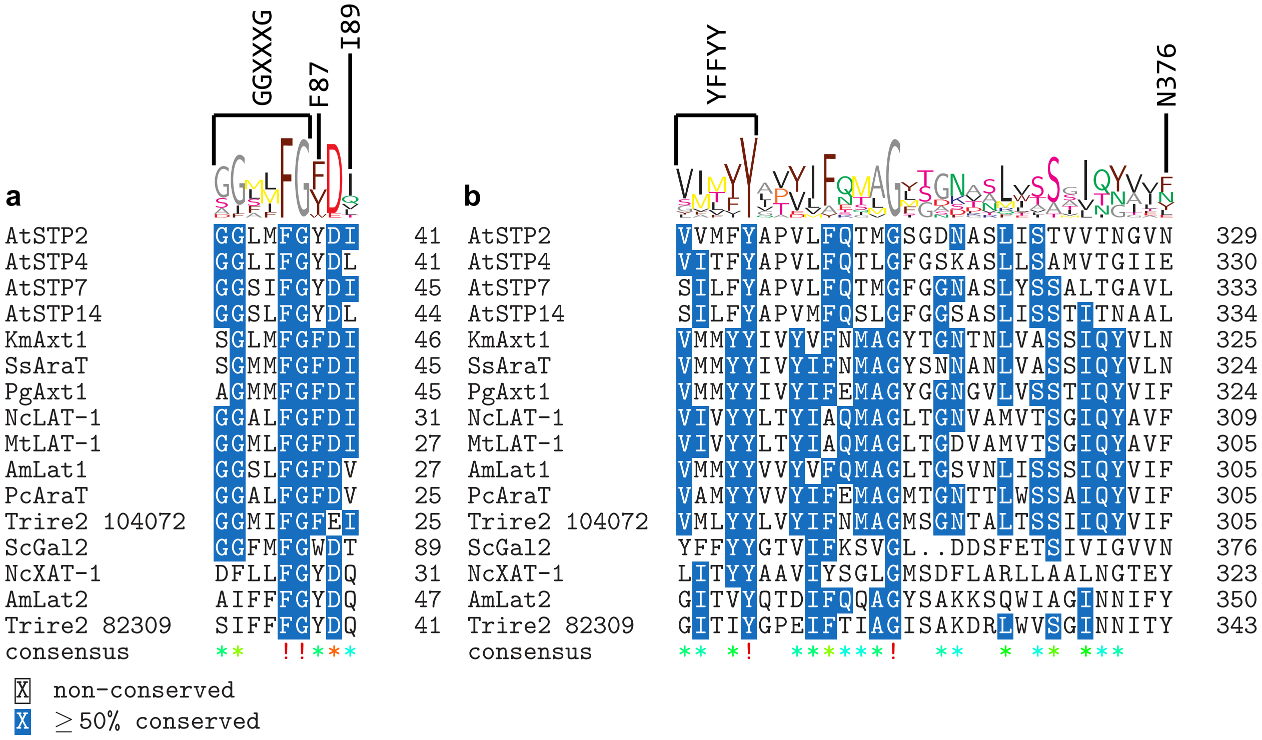

Figure S2. Subset of the multiple alignment of transporter amino acid sequences. **a** Important sequence determinants from the first TMD: `GGXXXG` motif, phenylalanine 87 and isoleucine 89 (Gal2 numbering). **b** Important sequence determinants from the sixth and seventh TMD: `YFFYY` motif and asparagine 376.

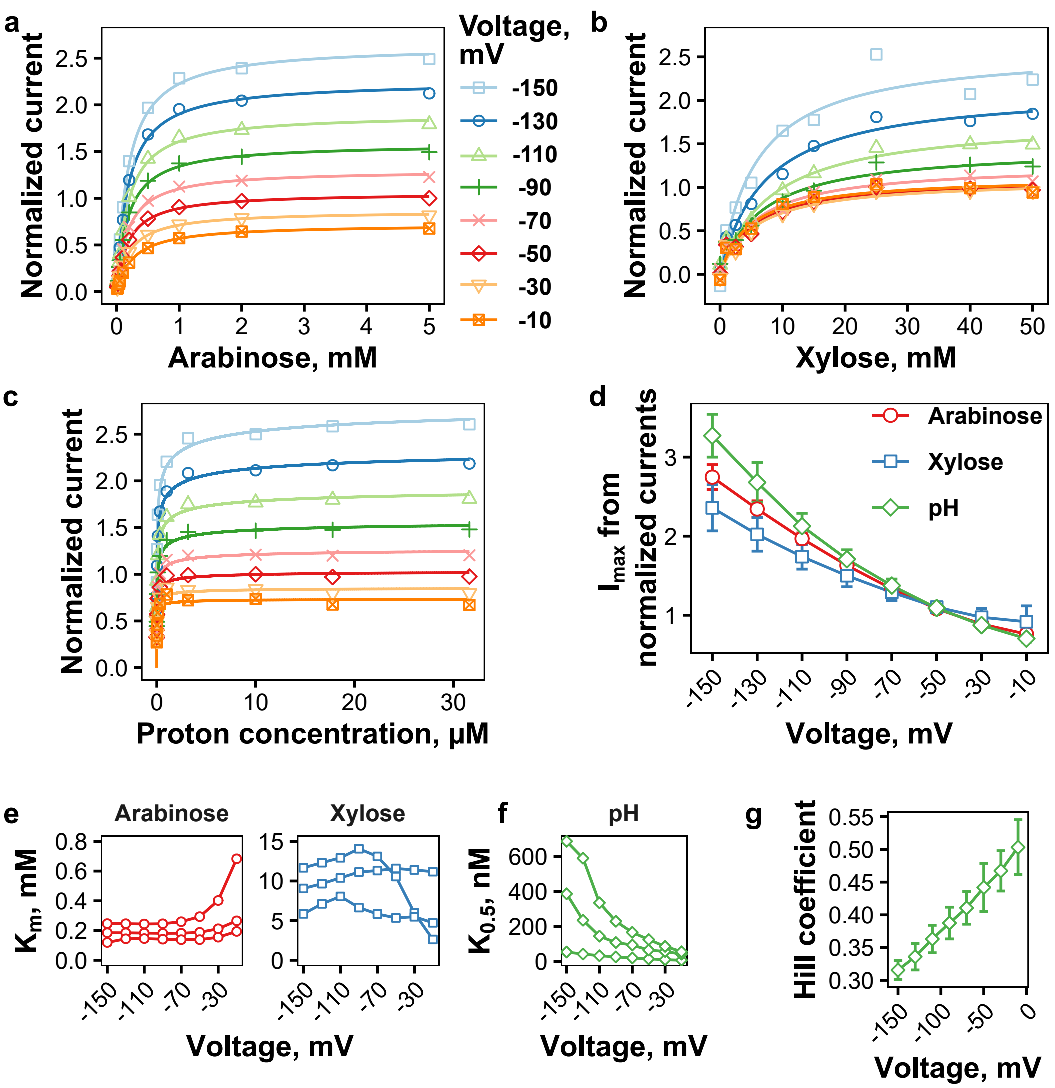

Figure S3. Analysis of voltage dependence of Trire2_104072 kinetics. a–c Normalized currents as a function of substrate concentration for L-arabinose (a), D-xylose (b) and protons (c) for each voltage are shown for representative experiments. Voltages are indicated in panel a. Currents as a function of proton concentration were measured in the presence of 5 mM L-arabinose. Values were normalized with the highest negative current obtained for each sugar at -50 mV. Michaelis-Menten (a, b) or Hill (c) kinetics predictions are shown as lines. Values obtained for voltages above 0 are not shown. **d** Dependence of I_{max} on voltage. I_{max} values predicted from panels a–c were plotted as a function of voltage. Error bars present standard deviation between three independent experiments. **e** Dependence of K_m on voltage for L-arabinose and D-xylose. Points and lines present results for individual experiments. **f** Dependence of K_m on voltage for pH. Presentation as in panel e. **g** Relationship between voltage and Hill coefficient. Error bars present standard deviation between three independent experiments.

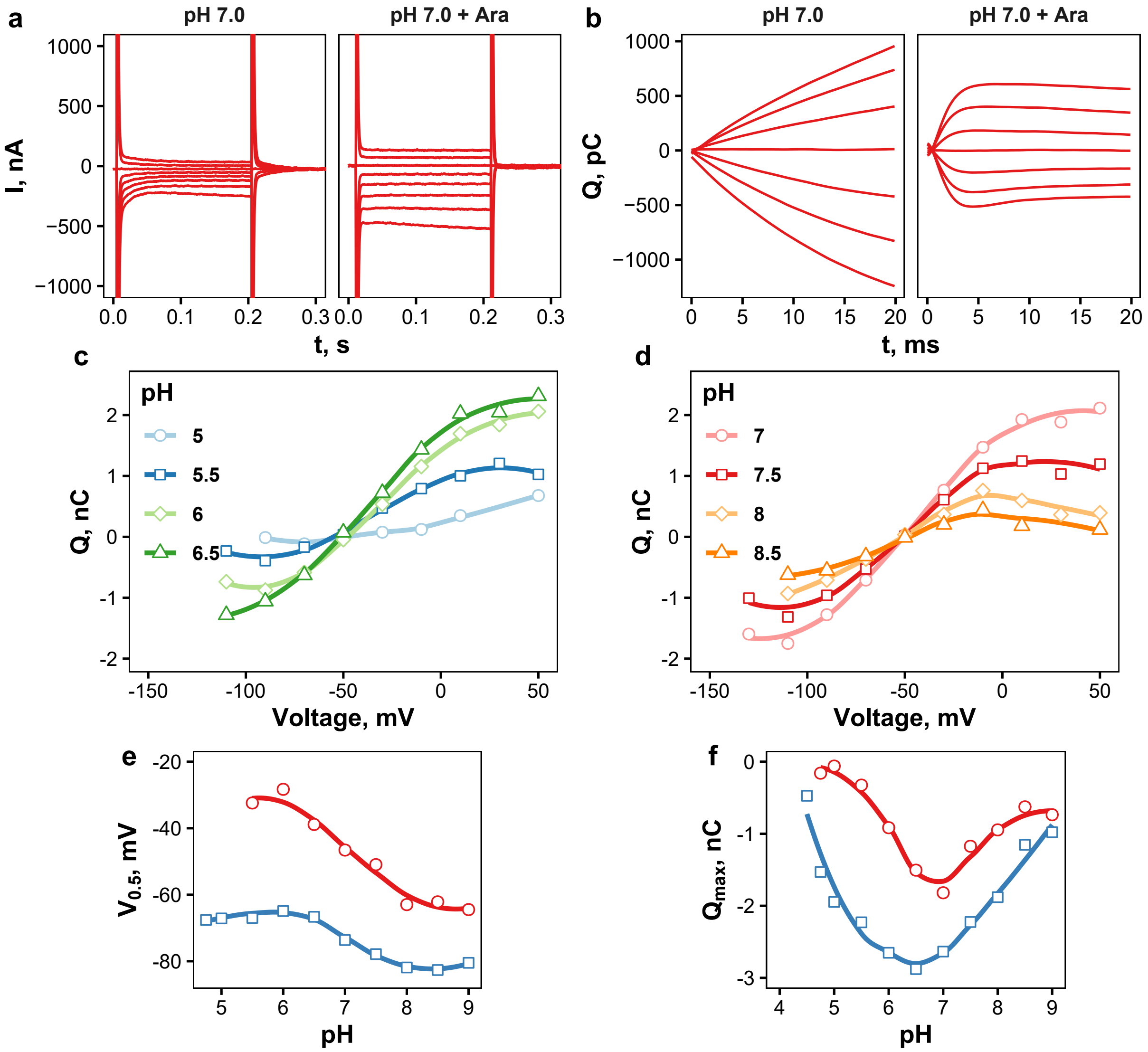

Figure S4. Analysis of pre-steady state kinetics of Trire2_104072. **a** Representative example of currents recorded in the absence and presence of 5 mM L-arabinose at pH 7.0. **b** Pre-steady state currents extracted from panel a. **c-d** Charge transfer as a function of voltage at low (c) or high (d) pH range. Representative results are shown. **e** $V_{0.5}$ as a function of pH. Results are shown for two oocytes, with the oocyte shown in panels a–d as red circles. **f** Q_{max} as a function of pH. Results are shown for two oocytes, with colors and shapes as in panel e.

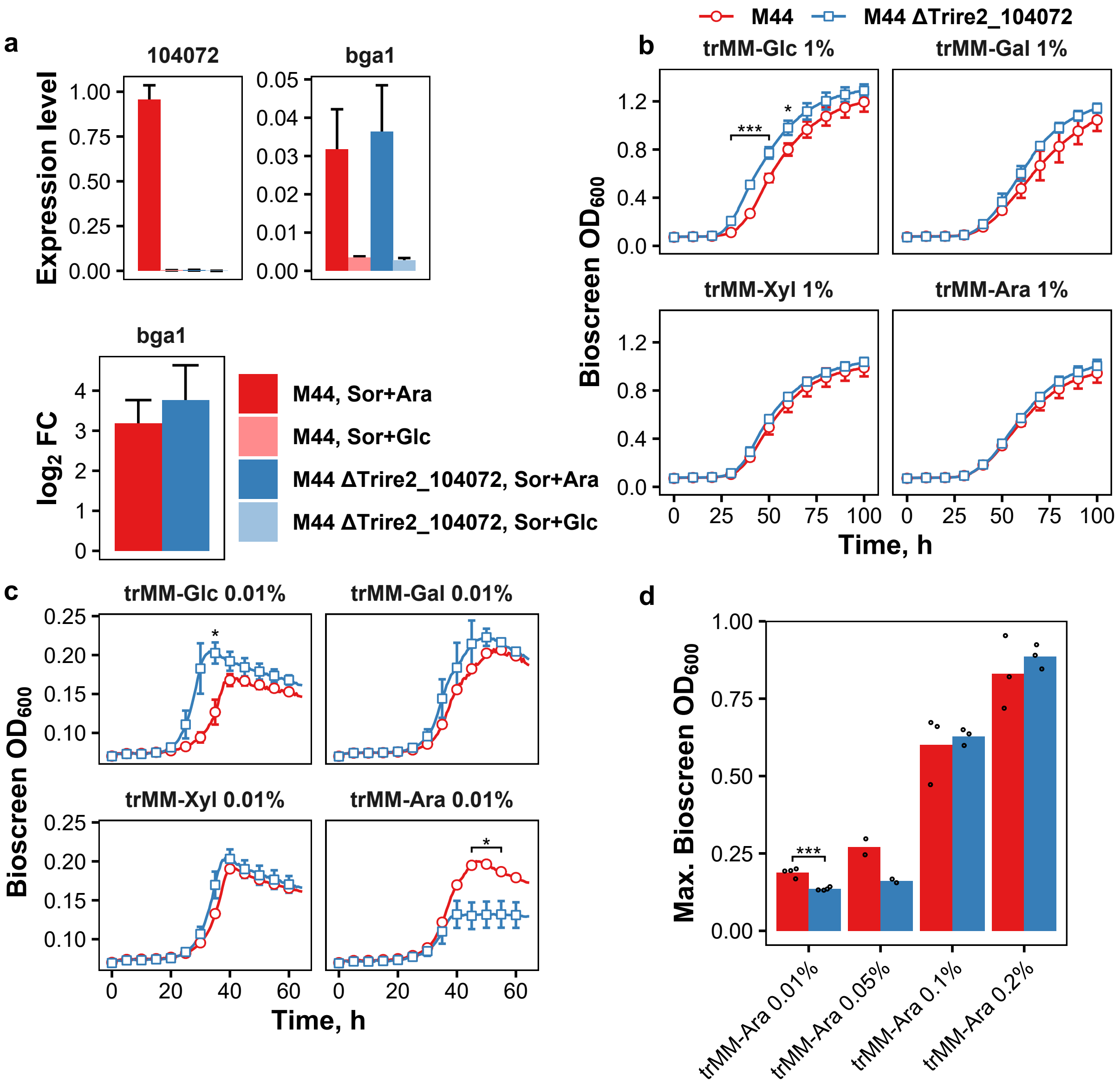

Figure S5. Characterization of the ΔTrire2_104072 mutant. **a** Expression levels ($2^{-\Delta C_p}$)of Trire2_104072 and *bga1* from sorbitol-grown cultures where L-arabinose or D-glucose was added to 1% concentration and RNA extracted 6 h later. Log_2 fold-changes between L-arabinose and D-glucose-added cultures are shown for *bga1*. *Sar1* and *gpd1* were used as the reference genes. Error bars present standard deviation between two biological replicates. **b** Growth curves of parental and ΔTrire2_104072 strain on minimal medium supplemented with indicated carbon sources. Error bars present standard deviation between 3 independent experiments. Error bars and points were drawn only for every 10 h for clarity. **c** Growth curves of parental and ΔTrire2_104072 strain on minimal medium supplemented with carbon sources from panel b in 0.01% concentration. Error bars present standard deviation between 2 biological replicates, and they and points were drawn only for every 5 h for clarity. Colors and shapes as in panel b. **d** Maximum OD_{600} values obtained from growth profiling experiments. Points present measurements from 2–4 independent experiments with bars presenting their mean, with colors as in panel b. Statistical significance in panels b–d was assessed with Student's *t*-test (*: $p<0.05$, ***: $p<0.005$).

References

1. Benocci T, Aguilar-Pontes MV, Kun RS, Seiboth B, de Vries RP, Daly P. ARA1 regulates not only L-arabinose but also D-galactose catabolism in *Trichoderma reesei*. FEBS Lett. 2018;592(1):60–70.
